# Recommendations for Improving the Modeling of Wintering Waterbird Population Sizes and Trends

**DOI:** 10.1002/ece3.72902

**Published:** 2026-02-17

**Authors:** U. Godeau, E. Gaget, L. Dami, K. Baddour, D. O. S. O. Daf, M. Dakki, T. Frost, M. Hornman, H. Kolberg, S.‐H. Lorentsen, B. Molina, F. E. F. F. Moniz, P. Defos du Rau

**Affiliations:** ^1^ Office Français de la Biodiversité, Tour du Valat, Le Sambuc Arles France; ^2^ Tour du Valat, Research Institute for the Conservation of Mediterranean Wetlands Arles France; ^3^ Parc National du Diawling ‐ Ministère de L'environnement et du Développement Durable Nouakchott Mauritania; ^4^ GREPOM/BirdLife Morocco Sale Morocco; ^5^ British Trust for Ornithology, The Nunnery Thetford UK; ^6^ Sovon Dutch Centre for Field Ornithology Nijmegen the Netherlands; ^7^ Ministry of Environment, Forestry and Tourism, Private Bag Windhoek Namibia; ^8^ Norwegian Institute for Nature Research Trondheim Norway; ^9^ Sociedad Española de Ornitología (SEO/BirdLife) Madrid Spain; ^10^ Instituto da Conservação da Natureza e das Florestas, IP (ICNF), Centro de Estudos de Migrações e Proteção de Aves (CEMPA) Lisboa Portugal

**Keywords:** biodiversity monitoring, imputation, International Waterbird Census, overdispersion, sampling design, spatial autocorrelation, zero inflation

## Abstract

Biodiversity monitoring at large spatial and temporal scales is essential for informing conservation policies. The International Waterbird Census (IWC) is one of the longest‐running global citizen science monitoring schemes, providing critical information to several international agreements. However, analyzing IWC count data poses statistical challenges, including zero inflation, overdispersion, spatial autocorrelation, and missing data. While various modeling approaches have been used to estimate waterbird population size and trends, their ability to handle these issues and the implications for trend estimates remain unassessed. Using IWC count data from five species in the East Atlantic Flyway, we compared four modeling approaches: TRIM (TRends and Indices for Monitoring data), LORI (Low‐Rank Interactions), and two generalized linear mixed models (GLMMs) with simple or optimized parametrizations. We benchmarked their performance in addressing zero inflation, overdispersion, and spatial autocorrelation across different realistic sampling designs (i.e., alternative dataset configurations). Our results highlight significant limitations in commonly used methods. Simple GLMMs, TRIM, and LORI generally failed to mitigate both zero inflation and overdispersion. In contrast, optimized GLMMs improved model convergence and better addressed these issues by selecting appropriate probability distributions. However, no single distribution performed consistently well across species and sampling designs. Spatial structures were effective in reducing spatial autocorrelation in most cases. We recommend a careful species‐specific selection of statistical methods when analyzing count data, as inadequate models may misrepresent population trends and thus misguide conservation efforts. Future research should explore the integration of advanced hierarchical and spatio‐temporal models to improve inference from large‐scale citizen science datasets.

## Graphical abstract

We assessed four statistical approaches (simple GLMM, optimized GLMM, LORI, and TRIM) for analyzing International Waterbird Census data and found that commonly used methods inadequately address zero inflation, overdispersion, and spatial autocorrelation. Optimized GLMMs improved performance compared to simple GLMMs, and LORI was a good candidate with further developments, but no single model worked consistently across species or sampling designs. Our findings highlight the need for species‐specific model choices to ensure reliable population trend estimates for conservation.

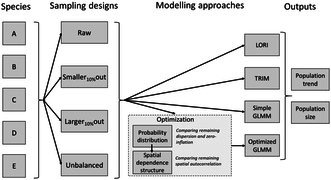

## 1 Introduction

Long‐term biodiversity monitoring schemes are useful to support management decision‐making and inform policy (Hughes et al. 2017). One of the longest‐running and most global biodiversity monitoring schemes is the International Waterbird Census (IWC) (Stroud et al. 2022), coordinated by Wetlands International. The IWC was launched in 1967 and today covers more than 25,000 sites in more than 100 countries, making it one of the largest standardized monitoring schemes based on citizen science (Sayoud et al. 2017; Amano et al. 2018). The IWC has allowed several sound and significant advances in ecology and conservation (e.g., Green and Elmberg 2014; Amano et al. 2018; Gaget et al. 2018). In particular, it is used to assess waterbird population size and trend, to inform international agreements and treaties, and support their policy decisions. It is central in supporting policy of the EU Birds Directive (2009/147/EC), the Ramsar Convention on Wetlands, the Convention on the Conservation of Migratory Species of Wild Animals, and especially the African‐Eurasian migratory Waterbirds Agreement (AEWA Secretariat 2013).

Providing population size and trend is therefore among the most important outputs of the IWC. However, methods for statistical estimation of both these parameters are still an area in progress (Buckland and Johnston 2017; Nagy et al. 2022), especially as large‐scale schemes based on citizen science may suffer from some additional statistical biases and challenges compared to structured, standardized monitoring programs conducted by trained observers (Kosmala et al. 2016; Johnston et al. 2021). Zero inflation (Zipkin et al. 2014; Tirozzi et al. 2022), overdispersion (Zuur et al. 2009), sampling design (Aubry et al. 2023), missing data (Atkinson et al. 2006; Dakki et al. 2021; Bowler et al. 2025), and spatial bias (Rocchini et al. 2023) are among statistical issues frequently affecting the estimation of wildlife population sizes and trends. The IWC abundance data are characterized by a large proportion of zero, a large variance in counts (both within and between species), and spatial dependence structures (e.g., Gaget et al. 2020; Folliot et al. 2022). Despite the IWC's objective of monitoring waterbirds annually, surveys are not carried out at every site each year, resulting in variation in the missing data across sites and years (Dakki et al. 2021). A substantial amount of missing data can jeopardize sound estimation of population size and trend by unbalancing sampling in time and space (Atkinson et al. 2006; Nakagawa and Freckleton 2008; Łopucki et al. 2022; Bowler et al. 2025). While some missing values result from random sampling imbalance, the main concern arises when data are not missing at random (MNAR). In such cases, missingness is related to the variable of interest, which can bias estimates of population trends. For example, systematically missing data from sites with very low or very high abundances may lead to over‐ or underestimation of temporal changes (Bowler et al. 2025).

All these issues, if not properly accounted in the modeling approach, potentially violate modeling assumptions and increase the risk of biased estimations or imprecise trend estimates. For example, inaccurate estimation of population trend or uncertainty can compromise the reliability of management and conservation decisions based on these data. While our study focuses on the IWC, the statistical challenges and methodological insights discussed here are relevant to national and international bird monitoring schemes worldwide, particularly for surveys occurring in the non‐breeding season when large bird flocks can be recorded, but also for breeding bird surveys such as the North American Breeding Bird Survey and the Pan‐European Common Bird Monitoring Scheme.

We hypothesized that some standard modeling approaches may not always succeed in dealing with these issues. We therefore benchmarked four modeling approaches using IWC count data from five waterbird species from the East Atlantic Flyway (EAF). We compared the modeling approaches in terms of their ability to deal with overdispersion, zero inflation (ZI), and spatial autocorrelation (SA). Two modeling approaches are specially designed for data imputation, namely LORI (Low‐Rank Interactions, Robin et al. 2019) and TRIM (TRends and Indices for Monitoring data, Van Strien et al. 2004). TRIM is the most widely used to report waterbird population sizes and trends to conservation policies (e.g., Nagy and Langendoen 2020), while LORI is relatively recent but performant for data imputation when the number of covariates is large (Dakki et al. 2021). In addition, we assessed two generalized linear mixed‐effect models with simple or optimized parametrization (with selection of the most appropriate probability distribution and incorporation of a method to account for spatial autocorrelation). Mixed‐effect models are commonly used and regularly improved to model biodiversity data (e.g., Bolker et al. 2009; Anderson et al. 2022). We assessed these four modeling approaches on four sampling designs (i.e., alternative dataset configurations) to evaluate model performance in a realistic range of scenarios sampling biases, reflecting challenges commonly encountered in large‐scale monitoring schemes (Thornton et al. 2011). These scenarios represented real‐life sampling patterns where sampling is biased towards smaller or larger sites, with the bias being either constant or varying over time.

## 2 Methods

### 2.1 International Waterbird Census Data

**FIGURE 1:**
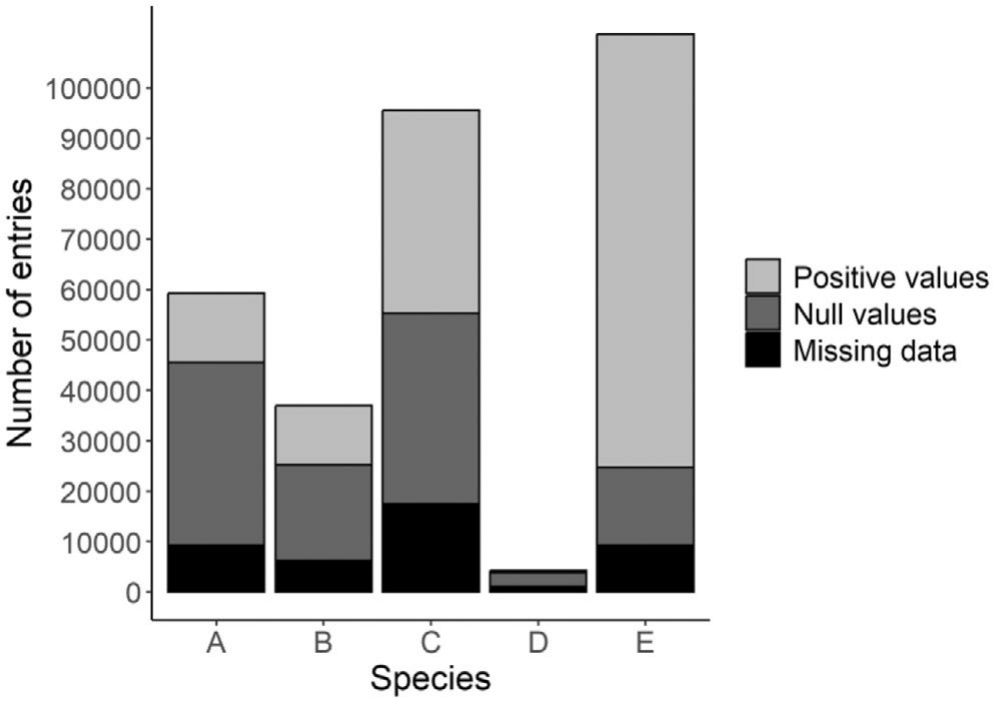
Total number of entries for the five waterbird species according to the nature of the data (positive count in light gray, null counts in dark gray, and NAs in black), after data filtering.

**FIGURE 2:**
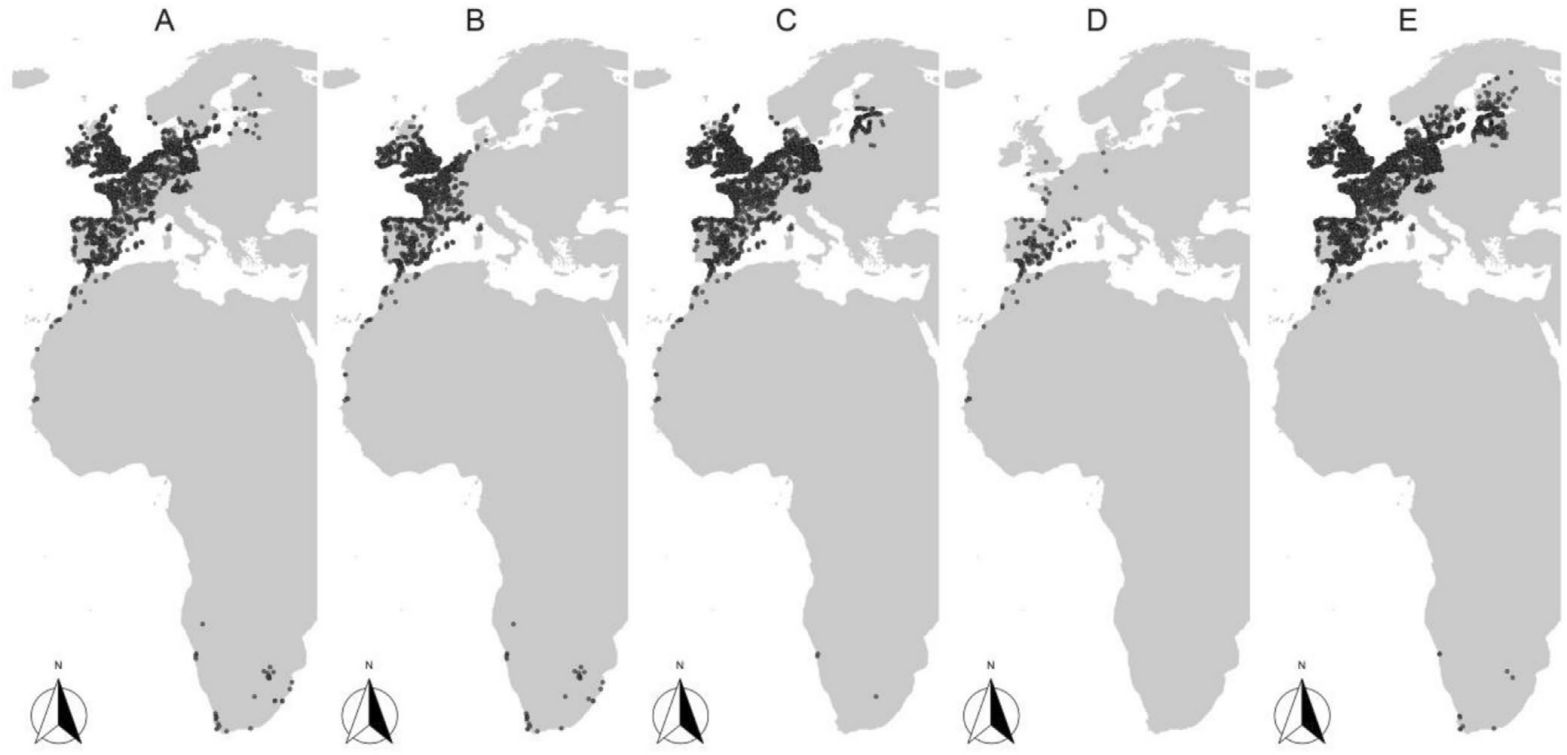
Monitoring sites for the five EAF species studied (plots A–E), after data filtering.

We used site‐ and year‐specific count data from five waterbird species from the EAF as examples and modeled their time‐series from 1995 to 2020 (Table 1, Figure 1). We chose to keep species identity undisclosed in the manuscript (coded from A to E) and to the authors involved in the modeling, in order to focus on statistical challenges rather than ecological inferences. These species can be any of the waterbirds wintering over the EAF, for example, ducks, waders, herons, etc. The IWC takes place once a year in mid‐January, under the terms of the Wetlands International (2010) Protocol. The EAF monitoring sites are distributed from Northern Europe to South Africa along the east Atlantic side of Europe and Africa (Figure 2). Zeros have been generated on a survey when the species was not observed, on sites where the species has been observed at least once over the monitoring period. Not all sites are monitored every year, resulting in a non‐negligible number of missing site‐year data (NAs). To limit the ratio of missing data, we focused on the 1995–2020 period, starting when most sites were already monitored and finishing before the lag effect in data reporting to the EAF database. We also filtered out sites with fewer than 15 surveys during the 26‐year study period, to decrease missing data to a reasonable ratio of less than 25% (Table 1; Dakki et al. 2021). In total, after filtering, we used count data for five species from 4858 sites (from 163 to 4792 sites per species) monitored over 26 years (Table 1).

**TABLE 1:** Description of the count data for the five waterbird species.

Species code	N sites	Mean count/site	SD count/site	Q5% count/site	Q50% count/site	Q95% count/site	Max count/site	N site. year	N NAs	% NAs
A	2280	52.9	623.6	0.0	0.0	130.0	45,300	59,280	9300	15.7
B	1423	16.3	148.8	0.0	0.0	48.0	6169	36,998	6284	17.0
C	3675	278.4	1802.3	0.0	1.0	921.0	96,000	95,550	17,488	18.3
D	163	2.6	31.8	0.0	0.0	5.0	1291	1291	1102	26.0
E	4792	341.8	1673.1	0.0	55.0	1180.0	85,576	85,576	23,186	7.5

*Note:* N sites gives the total number of sites where the species was counted at least 15 years; Mean, SD, Q5%, Q50% (median), Q95% and max count/site respectively give the mean, SD, quantile 5%, quantile 50%, quantile 95% and maximum counts per site and year; N site.year gives the total number of entries, NA included; N and % NAs respectively give the number and percentage of missing data (NAs).

### 2.2 Sampling Designs

Here, *sampling design* refers to the dataset configuration we analyzed (raw vs. subsampled datasets), rather than the procedure by which data were collected. We used four different sampling designs to mimic a range of sampling configurations that could occur in the field and affect the characteristics of the dataset.

First, we used raw IWC count data (hereafter “Raw”), only filtered according to the criteria above (i.e., study period, number of surveys per site).

Second, we removed the 10% sites with the highest mean counts from the raw data (hereafter “Larger_10%_out”). Thus, we simulate a realistic scenario in which the largest sites are too vast to be monitored. Because the probability of exclusion depends on the site's population size, these missing data are not missing at random (MNAR). This sampling design is expected to reduce overdispersion issues and lead to a smaller population size.

Third, we removed the 10% sites with the lowest mean counts from the raw data (hereafter “Smaller_10%_out”). This design simulates a realistic scenario in which smaller sites are considered not worth the sampling effort. Again, these exclusions generate MNAR data patterns, as missingness is dependent on population size. We expect less zero inflation and a larger population size.

Fourth, we simulated a strong temporally unbalanced sampling design (hereafter “Unbalanced”), by converting into NAs (i) 20% of the counts from sites with the highest mean counts (mean count > quantile 0.75 of mean counts) from 1995 to 2007, and (ii) 20% of the counts from sites with the lowest mean counts (mean count < quantile 0.25 of mean counts) from 2008 to 2020. This sampling design investigates a change in the allocation of monitoring resources over time from sites hosting small abundances to sites hosting large abundances. Because missingness depends on site abundance and changes over time, this scenario generates temporally varying MNAR data patterns. Such a mechanism can bias trend estimates, for example, by making population increases appear solely due to the changing proportion of high‐abundance sites. Such concerns are not unique to the IWC and may arise in other large‐scale monitoring designs facing similar shifts in site allocation (Fournier et al. 2019).

### 2.3 Modeling Approaches

**FIGURE 3:**
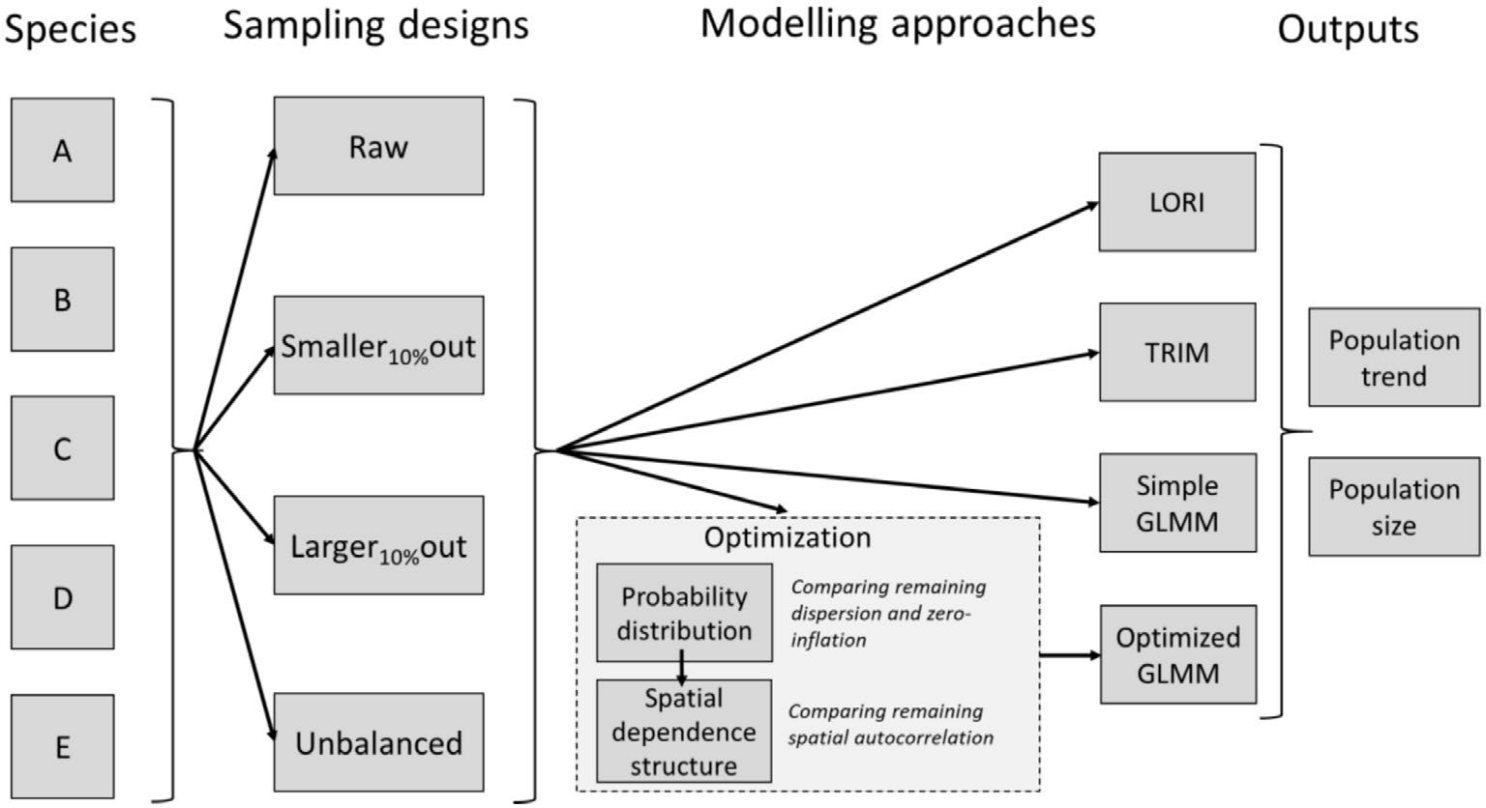
Schematic representation of the comparative modeling framework. Population trends and sizes of five species (A–E), under four sampling designs, are compared across four modeling approaches.

We used four modeling approaches to assess both population size and trend for all five species and all four sampling designs (Figure 3): (1) simple GLMM, (2) optimized GLMM, (3) TRIM and (4) LORI. We used the R package sdmTMB (Anderson et al. 2022) to fit the simple GLMM and optimized GLMM, because this package offers a large choice of probability distribution and allows for the use of different spatial structures (see below). For the GLMMs, we used the “sanity” function from the *sdmTMB* package to check the model fit, including convergence, variance–covariance matrix validity, parameter consistency, overfitting, multicollinearity, and residual distribution. We ensured that all sanity checks were passed, verifying the robustness and interpretability of our GLMMs, before further analysis.

#### 2.3.1 Simple GLMM

The simple GLMM corresponds to a standard modeling approach, in which waterbird abundance is fitted with a Poisson probability distribution. The year (numeric variable) is used as a fixed effect to assess the temporal trend (O'Hara and Kotze 2010). We added a random effect of site identity on the intercept to account for repeated samplings (Bird et al. 2014). The use of a simple GLMM with a site random effect is likely the first that an ecologist might do to analyze abundance data over time at repeated multiple locations, before adjustments for statistical issues (e.g., Zuur and Ieno 2021).

#### 2.3.2 Optimized GLMM

The optimized GLMM approach builds on the simple GLMM (with year as a fixed numerical effect and site as a random effect) but aims at adjusting the model parametrization to control as much as possible for overdispersion, zero inflation, and spatial autocorrelation (Legendre 1993; Dale and Fortin 2002). For each of the five species and the four sampling designs, we successively fitted GLMMs with different parametrizations to identify the probability distribution and then spatial structuration most fit to the data. We hypothesized that both probability distribution and spatial structuration could differ depending on species and sampling designs.

First, we selected the most adequate probability distribution based on overdispersion and zero‐inflation features among Poisson, Negative binomial type 1 (variance scaling linearly with the mean), Negative binomial type 2 (variance scaling quadratically with the mean), Tweedie, hurdle lognormal, hurdle Gamma, hurdle truncated negative binomial type 1, hurdle truncated negative binomial type 2, hurdle Poisson‐link Gamma, and hurdle Poisson‐link lognormal. This list follows an increasing order of statistical complexity and computation time. For instance, hurdle models (often called delta models) are composed of two parts, the first using a binomial or Poisson‐link distribution and the second typically using distributions for positive counts. We did not consider quasi‐likelihood approaches, despite their relevance to address over‐dispersion, as these were not implemented in *sdmTMB*. To assess overdispersion and zero inflation, we used the “testDispersion” and “testZeroInflation” functions, respectively, from the package *DHARMa* (Hartig 2022), with default setting (alpha risk = 5%). We selected the first probability distribution in order of appearance as listed above, for which overdispersion and zero inflation proved not significant. However, if all probability distributions returned significant overdispersion or zero inflation, we selected the distribution with the lowest overdispersion and zero‐inflation test values in the same priority order.

Second, after the selection of the probability distribution for each case, we compared different spatial structures to accommodate and mitigate potential spatial autocorrelation. The spatial autocorrelation (SA) is a frequent pattern in species distribution data, that is, locations close to each other exhibit more similar or dissimilar values than would be predicted by chance alone (Dormann et al. 2007). We successively used random intercept (RI), basis penalty smoothing (BPS), and Gaussian random field (GRF). For the RI, we build on the methodology presented by Folliot et al. (2022), by grouping sites to 100 × 100 km grid cells and adding the cell identity corresponding to each site as a random effect on the intercept. Thus, the spatial structure was included as a hierarchical level. For the sake of simplicity, we focused our tests on a single grid resolution that was appropriate for addressing spatial autocorrelation among wintering waterbird species (e.g., Folliot et al. 2022). However, it is important to note that other resolutions could have been explored. For BPS, we used a two‐dimensional smoothing function on site coordinates to model surface trends (Dormann et al. 2007). For GRF, we used the Stochastic Partial Differential Equations (SPDE) to approximate the Gaussian random field (Lindgren et al. 2011). The spatial mesh matrix needed to apply the SPDE approach was created from site coordinates using the package *sdmTMB*. To explore the effect of the mesh parameters on spatial autocorrelation mitigation, we used meshes, either unconstrained or constrained by the coastline as a physical barrier along the flyway. In addition, we compared the effect of minimal distance between mesh vertices, using a cutoff distance of 0.5 and 1.0 decimal degrees. For each model, we assessed Moran's I (Rangel et al. 2010) and autocorrelation with the “testSpatialAutocorrelation” function from the package *DHARMa* (alpha risk = 5%). We retained the model with the first spatial structure able to control for SA following the order: RI, BPS, GRF, and GRF with barrier, based on the potential computational complexity of the model.

#### 2.3.3 Trim

TRIM is based on a Poisson distribution that can be relaxed into a quasi‐Poisson distribution if specified to consider overdispersion ($\mathrm{Var}\left(Y\right)=\sigma^{2}\times \lambda$, with λ the mean of the Poisson distribution and *σ* = 1 for the Poisson distribution or *σ* being estimated for the quasi‐Poisson distribution). TRIM can also accommodate serial correlation to relax the assumption of independence over time, by estimating a correlation between adjacent time points: $\mathrm{cor}\left(f_{i,j},f_{i,j+1}\right)=\rho$.

In TRIM, only factor covariates are allowed (with a limited number of levels). Therefore, year was thus included in the model as a factor. To attempt accounting for spatial autocorrelation and because adding site coordinates would result in too many levels, we added four large geographical regions as factors: Northern Europe, Southern Europe, North Africa, and sub‐Saharan Africa (Table SI.1 for countries distribution into those four categories).

We implemented TRIM using the *rtrim* package (Bogaart et al. 2018) in two steps: (1) first, with the overdispersion and serial correlation options activated, (2) then, following warning messages intended for users, we relaunched TRIM, disabling the autocorrelation option if *σ* ^2^ < 1 and/or the serial correlation option if *ρ* < 0.2.

TRIM does not provide estimates of zero inflation, so we adapted an alternative approach to assess zero inflation. Specifically, we were unable to apply the DHARMa approach due to the absence of multiple imputations as required by DHARMa. Instead, we employed a chi‐squared test to compare the number of observed vs. expected zeros generated by TRIM. Expected zeros were derived from TRIM's fitted data, rounded to the nearest integer. This method evaluates whether the distribution of observed and expected zeros is consistent, providing a basic diagnostic tool for assessing zero inflation. However, we recognize that this approach has limitations and may not fully address the complexities associated with zero inflation (for a comparison of the two methods, see the Supporting Information S1). To estimate residual overdispersion in TRIM models, we used the approximate method based on the comparison (with chi‐squared test) of the sum of squared Pearson residuals and the residual degrees of freedom. This result is directly accessible in the goodness‐of‐fit chi‐squared outputs of the TRIM model summary. Moran's I coefficient and *p*‐value were computed using the “Moran.I” function from the *ape* package, based on site‐level differences between observed counts and model‐predicted means (Paradis and Schliep 2019).

#### 2.3.4 LORI

Like TRIM, the recent *lori* package aims at imputing large incomplete count datasets but can accommodate large predictors set due to the penalized approach of its relaxed Poisson model (Robin et al. 2019; Dakki et al. 2021). With LORI, only numerical covariates are allowed. Thus, we added year as a continuous fixed effect as well as site longitude and latitude to attempt accounting for potential spatial autocorrelation. We performed multiple imputations (*n* = 100, with the “mi.lori” function), to obtain the mean and variance of the imputed data for each site and year.

To estimate residual overdispersion for LORI model, we also compared the sum of squared Pearson residuals and the residual degrees of freedom. As this comparison is not directly available within the *lori* package, we calculated it based on the model outputs, as proposed by Bolker (https://bbolker.github.io/mixedmodels‐misc/glmmFAQ.html#overdispersion). To estimate the residual zero inflation, we applied the method implemented in the DHARMa package, comparing the observed number of zeros in the data to the expected number across multiple predictions generated from the model's simulated distributions. Finally, Moran's I coefficient and associated *p*‐value were also computed based on the model outputs, using the “Moran.I” function from the *ape* package.

### 2.4 Estimating Population Size and Trend Indexes

We estimated models' performance with the use of Relative Root Mean Squared Error (RRMSE). This metric is based on the square of the residuals and quantifies the discrepancy between observed and predicted values, giving more weight to large discrepancies. We then estimated population size and trend indexes from all four modeling approaches, five species, and four sampling designs. For all models—TRIM, LORI, and the GLMMs—we obtained direct estimates of mean population size on the original scale. We did not provide population size and trend when models returned a convergence issue.

For the simple GLMM and optimized GLMM, we used the “get_index” function (*sdmTMB* package) to assess population size index as the sum over all sites of the site‐specific abundances estimated per year, and its 95% confidence interval (95% CI). For TRIM and LORI, total population size per year was calculated as the sum of values estimated by the model, whether initially observed or missing. For TRIM, we extracted the “fitted” values of the time totals output. For LORI, the annual total population (total_pop_) was obtained by summing site‐level values across all sites for each year. Uncertainty was estimated using Rubin's rules applied to the 100 multiple imputations, which accounts for both the variability between imputations and the variability within each imputation. The resulting total variance (Vtotal_pop_) was then used to calculate 95% confidence intervals (95% CI) for the annual population estimates as:

$${\text{Vtotal}}_{\mathrm{pop}}=W+\left(\;1+\frac{1}{m}\right)\times B$$

$95\%\mathrm{CI}={\text{total}}_{\mathrm{pop}}\pm 1.96\times \sqrt{{\text{Vtotal}}_{\mathrm{pop}}}$ where 1.96 is the 97.5th percentile of the standard normal distribution and

- *m* = number of imputations (here 100).
- *B* = variance between imputations.
- *W* = within‐imputation variance.

The trend in population size was estimated using a linear regression model, with estimated population size as the dependent variable and time as the predictor. The slope estimate represents the expected absolute annual change in population size. To facilitate comparisons across populations of different sizes, slopes were also standardized per 1000 individuals. Corresponding 95% confidence intervals were calculated accordingly.

## 3 Results

### 3.1 Selection of the Optimized GLMM

**FIGURE 4:**
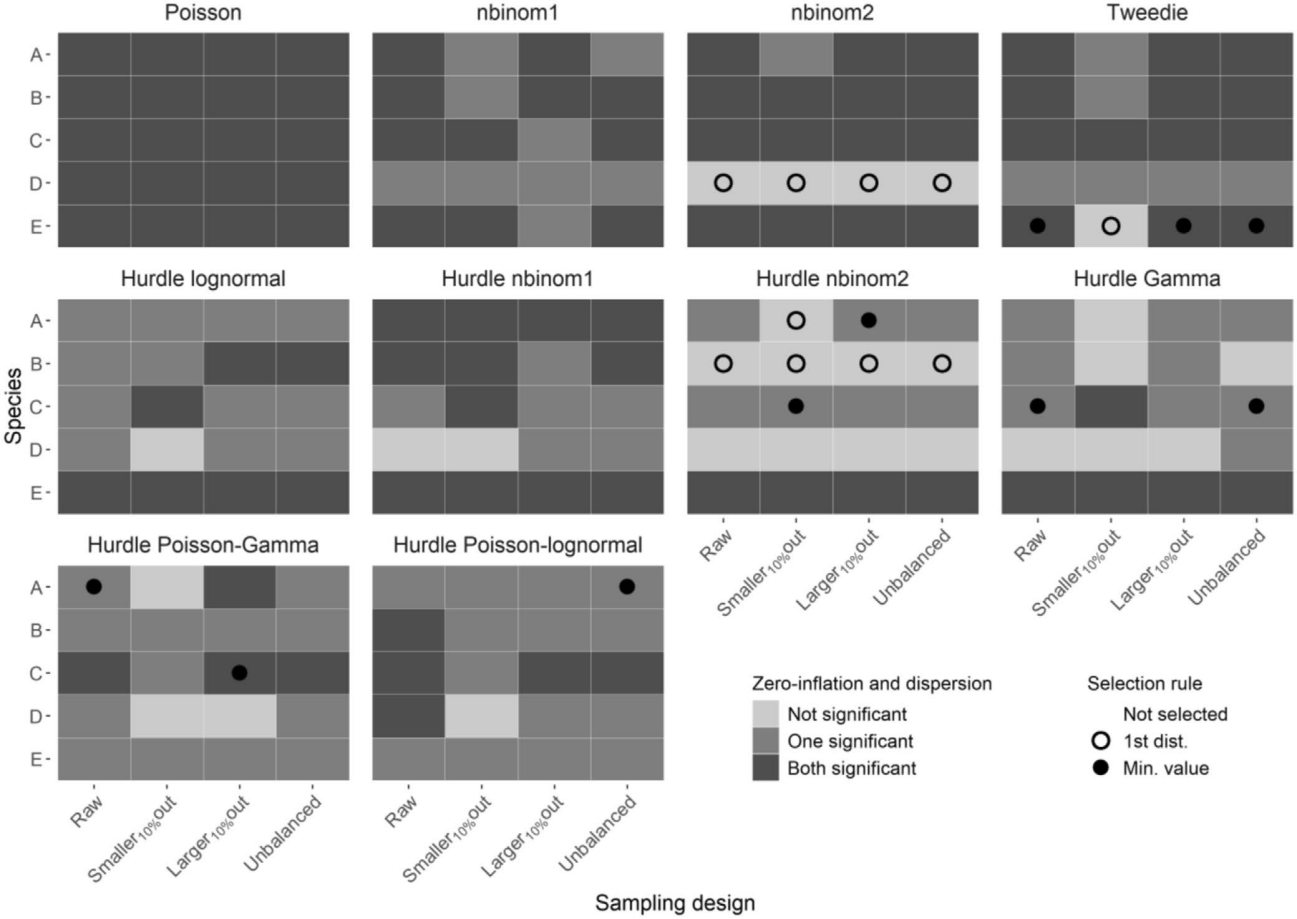
Selection of the best probability distribution in optimized GLMMs for the five species (A–E). The significance of zero inflation and/or overdispersion is indicated by the color of the tile, while the selection process of the proper distribution for each sampling design is indicated by a circle in the middle of the tile. When zero inflation and overdispersion were both not significant (“Not significant”), we selected for each sampling design the first probability distribution (“1st dist”) in order presented in the methods. If all probability distributions returned significant overdispersion or/and zero inflation (“One significant” and “Both significant”), we selected for each sampling design the distribution with the lowest overdispersion and zero‐inflation test values (“Min. value”) in the same priority order as before (see Section 2).

Significant residual zero inflation or overdispersion was found in 90% of the GLMMs. The selected probability distributions differed between species and sampling design (Figure 4). For almost every sampling design and species, the Poisson distribution performed the worst, as it enforces the restrictive mean–variance relationship and cannot capture the excess of zeros or variance relative to its assumptions (Figures SI1–SI5). No significant residual zero inflation or overdispersion was detected for the selected probability distributions in approximately 40% of the optimized GLMMs (Figure 4, black circles). Negative binomial 2 and hurdle negative binomial 2 were selected in most of these cases, and to a lesser extent Tweedie and hurdle Gamma. When the selected probability distribution had the lowest residual overdispersion and zero inflation test values still significant (Figure 4, black dots), the preferred distributions were mostly Tweedie, hurdle Gamma, and hurdle Poisson‐Gamma, as well as hurdle lognormal and hurdle Poisson‐lognormal on one occasion each.

**FIGURE 5:**
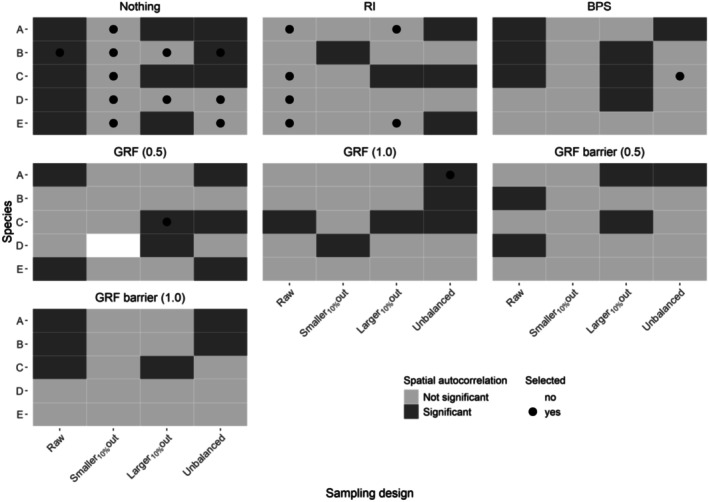
Selection of the best spatial autocorrelation setting in optimized GLMMs for the five species (A–E). The significance of residual spatial autocorrelation is indicated by the color of the tile, while the selection process is indicated by a dot in the middle of the tile. When residual spatial autocorrelation was significant, we selected the first setting in order presented in the methods. If all probability distributions returned significant residual spatial autocorrelation, we selected the distribution with the lowest residual spatial autocorrelation test values in the same priority order as before (see Section 2).

Spatial autocorrelation showed significant variability among sampling designs and species, with each spatial structure effectively removing spatial autocorrelation in more than half of their applications (Figure 5, Figures SI6–SI10). However, each spatial structure was rarely able to consistently remove spatial autocorrelation among all species and all sampling designs. For two cases (species C “Larger_10%_out” and species A “Unbalanced”), all the methods resulted in residual spatial autocorrelation. For the “Smaller_10%_out,” nothing was needed to make spatial autocorrelation not significant. On two occasions for this “Smaller_10%_out” (out of the five species and six methods), adding a spatial structure resulted in significant spatial autocorrelation (species B method RI; species D method GRF 1.0), and on one occasion the model could not converge (species D method GRF 0.5). Models with complex spatial structures (GRF and GRF barrier) were rarely, if ever, selected, as simpler models were chosen instead. However, these complex models were still regularly able to mitigate spatial autocorrelation (Figure 5).

### 3.2 Modeling Approaches Performance

**FIGURE 6:**
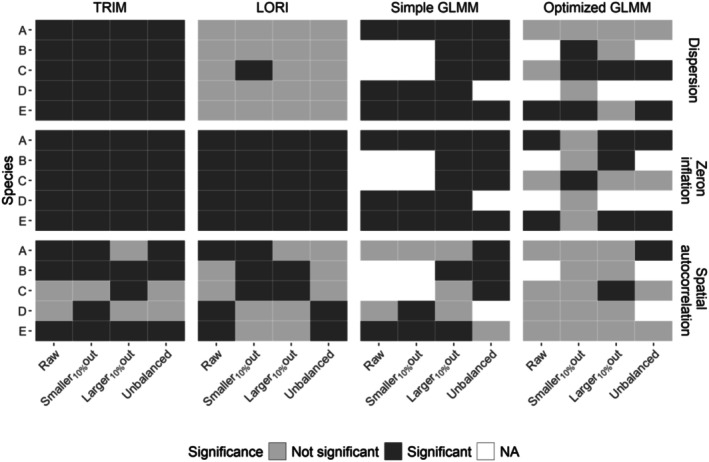
Test of the residual zero inflation, overdispersion, and spatial autocorrelation in LORI, TRIM, simple and optimized GLMMs approaches on the five species (A‐E) and the four sampling designs. For GLMMs, when models failed to converge, the cell was left blank.

Simple GLMM, TRIM, and LORI always failed to mitigate both zero inflation and overdispersion (Figure 6) and had significant but limited spatial autocorrelation in most of the models (−0.1 < Moran's I < 0.12, Figure 6, Figures SI11–SI15). Compared to simple GLMM, optimized GLMM allowed for an effective increase in convergence (from 15 non‐converging models to six) and reduced or removed residual zero inflation and/or overdispersion (11 models still presented both issues compared to 30 in the simple GLMM). All selected optimized GLMMs but three had no significant spatial autocorrelation (Figure 6). In addition, simple GLMMs exhibited more convergence issues compared to optimized GLMMs (white cells in Figure 6), meanwhile, both TRIM and LORI showed no signs of nonconvergence (according to the quality control checks implemented within these approaches). Adding a spatial structure to the model tended to affect zero‐inflation and/or overdispersion in the optimized GLMMs: in 17.8% of cases it was reintroduced compared with the corresponding simple GLMM, while in 8.9% of cases it was reduced, depending on the dataset (e.g., Figure 4 compared with Figure 6). Adding a spatial structure to the model also tended to undo convergence (13.3% of cases).

**FIGURE 7:**
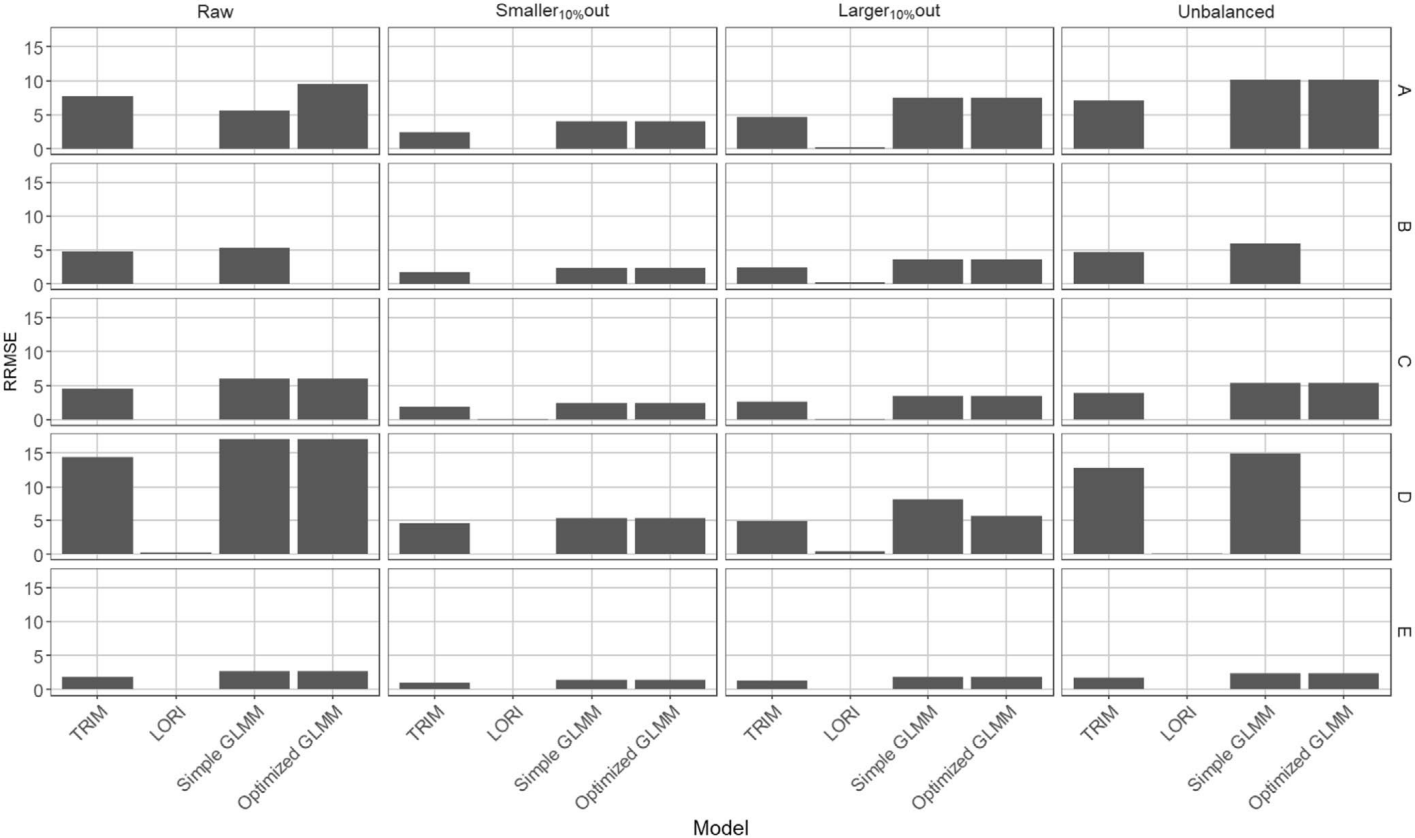
Relative root mean square error of final models for each sampling design and each species (A‐E).

In all sampling designs and for all species, LORI showed the best performance in terms of RRMSE, followed by TRIM and, lastly, the two GLMMs (Figure 7).

### 3.3 Population Size and Trend Indexes Among Modeling Approaches

**FIGURE 8:**
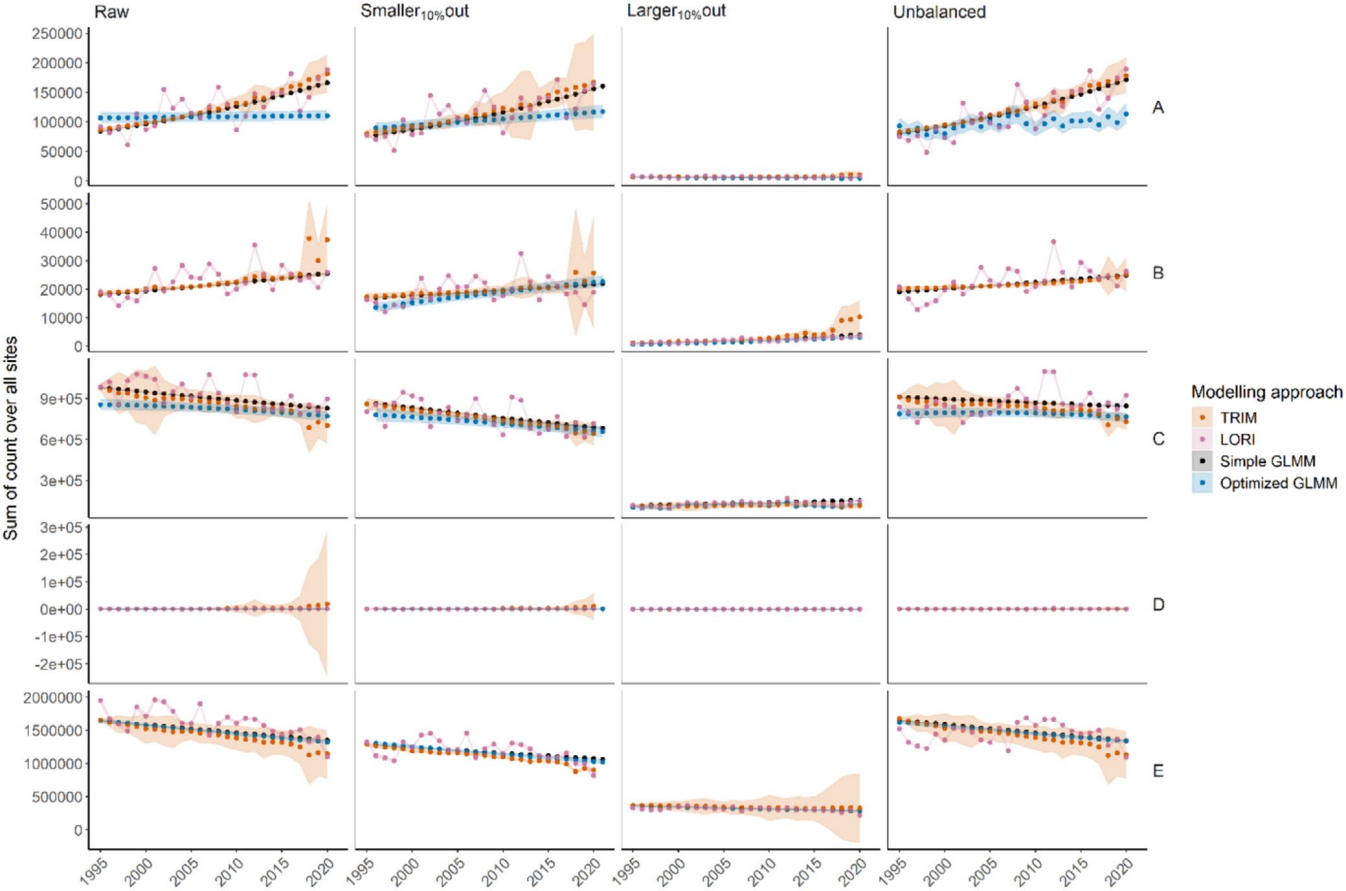
Yearly variations (and 95% CI) for all species (rows, A‐E) population sizes according to four sampling designs (columns) and five modeling approaches (colors).

**FIGURE 9:**
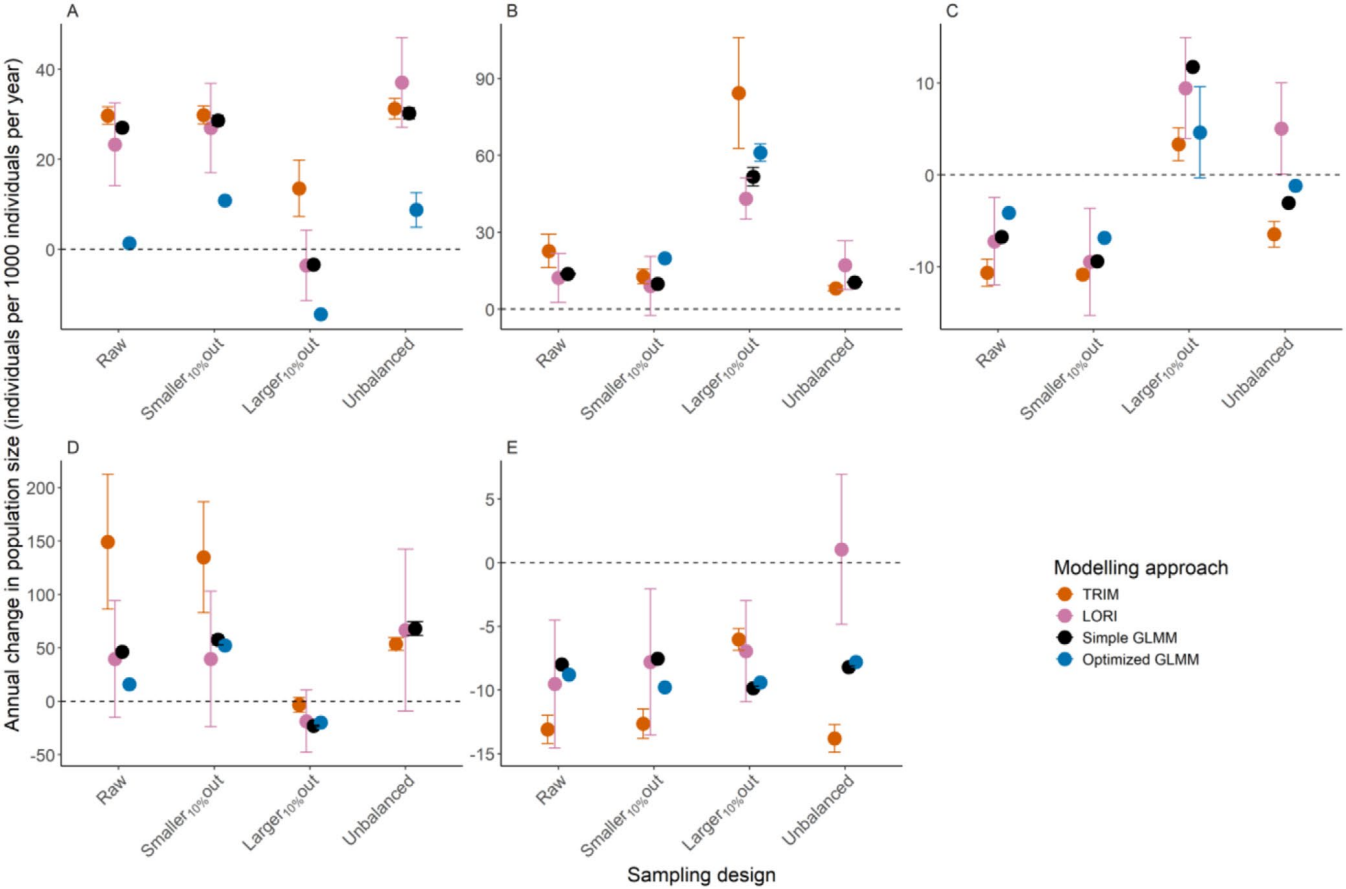
Estimated annual change in population size per 1000 individuals (with 95% CI) across sampling design for each species (A‐E) and modeling approach (colors).

The four modeling approaches differed in the interannual variability in estimated population size (Figure 8). For a given species and sampling design, estimated population size was of similar order of magnitude between modeling approaches. However, estimations from LORI, and to a lesser extent TRIM, exhibited larger interannual variations in population size than in simple and optimized GLMMs, in which estimated population sizes were distributed linearly (except when a spatially explicit GRF was used in optimized GLMMs for example, species A “Unbalanced,” Figure 8). The 95% CI were usually larger with TRIM than any other models and very small with LORI and with simple GLMM. Differences in population trends resulted in substantial differences of estimated population sizes (e.g., Figure 8, species A). For example, in 1995 with “Raw” sampling design, species A's population size was estimated around 106 k with the optimized GLMM and around 95k with LORI (~10.4% difference). However, in 2020, the estimates diverged significantly: approximately 110 k with the optimized GLMM and 185k with LORI (~68.2% difference), leading to markedly different trend estimates—nearly stable for the optimized GLMM and strongly positive for the other approaches (Figure 9).

Modeling approaches resulted in different trends for the same species and the same sampling design, except for species D with “Unbalanced” and “Larger_10%_out” sampling designs (Figures 8 and 9). Differences in trend magnitude and even direction between modeling approaches were found in most species, including for the “Raw” sampling design (such as in species A, see above) or species C with “Unbalanced” sampling design, where LORI returned a large positive trend, TRIM returned a large negative trend, while simple and optimized GLMMs returned small negative or nearly stable population trends (Figure 9). The 95% confidence intervals of the trend were generally smaller in optimized GLMM and simple GLMM than in LORI and TRIM.

### 3.4 Population Size and Trend Indexes Between Sampling Designs

For a given species and a given modeling approach, population sizes and trends also were not consistent between sampling designs, as the same approach could yield a strong positive trend in one case and a negative trend in another, indicating that the bias was not always in the same direction (Figures 8 and 9). For example, for species C, LORI returned a positive trend for the “Unbalanced” and “Larger_10%_out” sampling designs but a negative trend for the raw and “Smaller_10%_out” sampling designs (Figure 9C). For all species and modeling approaches, the “Smaller_10%_out” sampling design did not seem to modify the estimated population trends or sizes, as compared to the “Raw” sampling design, probably due to the small population fraction removed. In contrast, for all species, most models fitting the data from the “Larger_10%_out” sampling design led to much smaller population sizes and different trends compared to the other sampling designs. Removing the larger sites (“Larger_10%_out”) therefore has the most impact on trend estimation, followed by unbalancing the sampling design in time (“Unbalanced”), which affects the size and trend estimation for at least three of the five species.

## 4 Discussion

Estimating waterbird population size and trend is a primary goal of the IWC, but as shown in our study, methodological choices can significantly affect estimations. Using five undisclosed species monitored over the whole East‐Atlantic flyway to evaluate four methodological approaches on four sampling designs, our results showed that dealing with departures from model assumptions—such as excess zeros, overdispersion, and spatial autocorrelation—is not trivial. While TRIM and LORI have been used to inform waterbird population sizes and trends, our results suggest that they can violate statistical assumptions. Both often return outputs similar to a simple GLMM, for which violation of several statistical assumptions is shown to greatly influence estimations. Importantly, TRIM resulted in large uncertainty around estimations compared to optimized GLMM. Therefore, we recommend considering the statistical challenges addressed here when estimating population trends of any waterbird species. Based on their proportion of zero, count data distribution, and spatial autocorrelation, IWC count data may require different modeling parametrizations depending on species. We focused this work on time trend estimation and population size, although we acknowledge that studying population size would benefit from adding environmental covariates (Dakki et al. 2021) and more in‐depth knowledge of species‐specific detectability (Johnston et al. 2018). Although imperfect detection was not addressed in our study, it remains a key source of bias in wildlife monitoring (Nichols et al. 2000; Kellner and Swihart 2014) with implications for both inference and policy (Moore and Kendall 2004). Several methods exist to mitigate it, including double sampling (Pollock et al. 2002; Bart et al. 2004) and the use of observation covariates such as survey effort and observer expertise (Williams et al. 2002; Dénes et al. 2015; Johnston et al. 2018), and these should be more systematically integrated into IWC data analyses (Sanz‐Pérez et al. 2020).

In addition, we acknowledge that the “Raw” IWC datasets themselves may already be affected by sampling biases. These are difficult to evaluate not only because species identities are obscured, but also because, in practice, basic field data collection under the IWC protocol provides little information useful to address such biases (e.g., single count event, missing data on observation efforts). Consequently, our manipulations (e.g., excluding the smallest 10% of sites) may either compound or counteract pre‐existing biases, thereby amplifying or attenuating the intended treatment effect. Although it is not possible to disentangle these potential issues in the present framework, we recognize that further improvements should be brought to the IWC methodology at least to estimate and correct sampling biases.

### 4.1 Optimizing Models

Correctly specifying GLMM on IWC data is an essential step in accurately estimating population size and trend while minimizing potential biases due to data structure (Popovic et al. 2024).

High proportions of zeros are commonly observed in biodiversity count data (Dénes et al. 2015). Although widely used in count models, the Poisson distribution was the least efficient at fitting data with many zeros. Some non‐hurdle distributions may be sufficiently zero‐inflated to adequately fit waterbird count data like the Negative binomial and Tweedie distributions; however, the large quantity of zeros in IWC in general advocates for the use of hurdle models. Following Tirozzi et al. (2022), we recommend a careful selection of response distributions for IWC count modeling and advocate for expanding distribution choices in models like TRIM and LORI. While both have been used on IWC data (Dakki et al. 2021; Nagy et al. 2022), they only model Poisson‐distributed counts, which may not be suitable for zero‐inflated data. The reliance on the Poisson distribution in these imputation methods may decrease their capacity to predict zeros and could lead to overestimating population size and incorrect trend estimation. This bias would be even larger with an unbalanced amount of missing counts over time (Figure 9, “Unbalanced”). Unbalanced monitoring efforts are common in wildlife monitoring and are also observed in countries with decreasing IWC monitoring capacities. Therefore, such overestimation biases induced by methodological choices might have different effects between local differences in monitoring capacities.

Not a single method to account for spatial autocorrelation resulted in perfectly removing this issue. The simple GLMM was never able to correct for spatial autocorrelation when applied to “Raw” sampling design, while returning spatial autocorrelation in about 40% when used on the other sampling designs. Conversely, the use of RI in optimized GLMMs was efficient in the case of “Raw” sampling design, as suggested by Folliot et al. (2022). Our results show that GRF parameters can influence its ability to deal with spatial autocorrelation. Overall, TRIM and LORI poorly mitigated residual spatial autocorrelation, despite the inclusion of spatial variables in both models (coordinates in LORI, large geographical regions for TRIM). It is noteworthy that none of the spatial models used in this work integrated environmental predictors besides latitude, longitude, and year. However, one other solution to reduce spatial autocorrelation is to collect and integrate relevant environmental covariates into population models (Dormann et al. 2007). While incorporating such covariates is valuable and relies on species‐specific biological knowledge (Gaget et al. 2025), it does not by itself resolve potential biases inherent in the data, which we argue should be accounted for regardless. In practice, we recommend considering more extensively RI or GRF while analyzing IWC data (facilitated by the development of, for example, *mgcv* and *sdmTMB*; Wood 2011; Anderson et al. 2022) and to include meaningful environmental predictors that can both explain spatial dependencies and can contribute to impute missing data (Zuur and Ieno 2021). LORI, being able to incorporate a large number of numerical covariates, could also benefit from additional RI and GRF developments. The inclusion of covariates is widely recommended and can provide substantial benefits. Nonetheless, it is essential to remain mindful of the inherent limitations and potential risks associated with this approach: (1) at large spatial scales, obtaining relevant predictor data systematically across all sites is seldom feasible due to limited information on monitoring sites; (2) access to habitat polygons that would allow accounting for the extent and variability of ecological covariates is generally lacking; and (3) particular caution is required to avoid the introduction of additional sources of bias (Graham 2003; Kim and Shin 2016).

### 4.2 Different Modeling Approaches Can Produce Dissimilar Outputs

Our work highlighted that population trend and, to a lesser extent, size of a given taxon can sometimes differ markedly depending on modeling approaches, even if they are advocated as particularly suited to these types of population analyses. This calls for enhanced investment and consensus in methodological research as those metrics (population size and trend) are largely used to establish and guide national and international public policies. TRIM showed the most discrepancy for the species with the highest proportion of missing data (species D, Table 1) whereas LORI performed consistently with GLMMs for this species. Indeed, TRIM is known to accommodate relatively small proportions of missing data only (Nagy et al. 2022), contrary to LORI (Dakki et al. 2021). Both LORI and TRIM showed larger uncertainties compared to optimized GLMM, providing the most precise trends. Trend estimates also differed markedly between TRIM and LORI. We argue that all these discrepancies among TRIM, LORI, and GLMMs could be problematic in a conservation policy perspective, for example, to reliably assess conservation status. It should be noted that LORI is particularly designed to integrate a large number of environmental covariates to predict waterbird counts (Dakki et al. 2021) and would probably perform better than in this study through inclusion of more environmental variables (e.g., habitats, climate).

The limited annual variation in the fits of the optimal GLMM may be a consequence of the model structure. GLMs, by design, assume a linear annual trend, which may not fully capture interannual fluctuations in population size. However, they remain effective in identifying overall trends and provide a straightforward approach for reporting purposes and fitting management and policy decision‐making needs. In our study, we found that incorporating the mesh structure introduced more interannual variation in the GLMM fits, suggesting that spatial modeling can influence how temporal patterns are captured. While nonlinear models, such as GAMs, can provide a more detailed understanding of interannual variation, they were not considered here because the primary aim was to produce clear and interpretable trend estimates. If finer‐scale interannual variation is of particular interest, alternative approaches could be considered, such as using GAMs, which allow for more flexibility in capturing nonlinear trends, or refining the GLMM by incorporating a random slope effect at the site level to account for local variations in population dynamics.

One significant limitation of this study is the lack of comprehensive tools to assess whether the results of TRIM and LORI models were coherent and interpretable with established quality checks, compared to GLMMs. Consequently, comparisons between GLMMs and these alternative models may be biased in disfavor of GLMMs, as they underwent more rigorous assessments. Some TRIM and LORI models might fail to meet the quality criteria established for GLMMs, potentially affecting the robustness of their results. Developing comparable evaluation frameworks for models such as TRIM and LORI would enhance the fairness of such comparisons and improve their reliability and confidence in their outputs. Although optimized GLMMs might explicitly account for key structural properties of the data—such as zero inflation, overdispersion, and spatial autocorrelation—it do not achieve the lowest RRMSE. Structurally well‐specified models tend to propagate more sources of uncertainty, leading to wider and more variable predictive distributions. In contrast, models like LORI produce smoother predictions and generate many small nonzero values. These values lie close to the observed responses, which mechanically results in a substantially lower RRMSE even though the model fails to reproduce the true prevalence of zeros. In other words, very low RRMSE achieved by LORI indicates that it is highly effective at approximating the continuous part of the distribution even if systematically under‐represents structural zeros. Importantly, this suggests that LORI is already a strong candidate for generating imputed data, since it captures the main signal and yields values close to the observed magnitudes. In addition, the low RRMSE could also stem from the fact that LORI was the only tested model that allowed for interannual variation. By removing linear constraints on the predictions, LORI may produce more realistic estimates, which provides an additional argument in favor of using non‐linear models to predict missing data. In conclusion, LORI would benefit further development to incorporate a dedicated zero‐inflation component or to adapt its latent structure to better reflect the zero‐generating mechanism. If successful, such development could improve LORI's predictions and allow its use for realistic data generation, especially in applications where the accurate reproduction of both zero patterns and value distributions is required.

While it is not surprising that different statistical methods may lead to different results, our findings suggest that these discrepancies are often linked to violations of model assumptions when applied to ecological count data. These discrepancies should ideally be avoided in order to increase the chances of getting closer to the actual estimate and thus support the most accurate inference or management decision. In the context of bird monitoring, such violations are not incidental but rather inherent to the data, for instance due to imperfect detection, large flocks, or spatial and temporal heterogeneity. This indicates that methodological development should be done to address the most frequent sources of assumption violations in these datasets and to provide user‐friendly information on model quality. Overall, we recommend building more flexible and sophisticated modeling frameworks for analyzing IWC data that can be adjusted to species‐specific characteristics (e.g., abundance data distribution) and deal efficiently with statistical issues. In this perspective, discrepancies between methods should not be seen merely as inconsistencies, but as signals that highlight where current approaches fail and where more robust modeling frameworks are most needed.

### 4.3 Investing Extra Effort in Sampling Most Important Sites, That is, Those With Higher Counts

In our study, we investigated the consequence of a trade‐off between monitoring sites with and without the largest abundance. Population declines being best identified when species abundance values are high (Ficetola et al. 2018), it seems interesting to focus on sites with high counts when resources are limited. In our study, for four out of the five studied species, removing sites with higher counts had the greatest influence on the estimation of the population trend and/or size compared to the “Raw” data. Consequently, depending on the species, most of the necessary information on trends might lie in the sites with higher mean counts. We acknowledge that the use of IWC data is not limited to estimating population size and trend; much valuable information for science and conservation can be found in smaller sites (e.g., diversity, habitat connectivity), which therefore should not be discarded. However, a recommendation for IWC monitoring would be to ensure that wetlands important for waterbirds species are a priority for regular monitoring (Sayoud et al. 2017), including in Africa. Because sites with higher counts could be the most important in driving trends, we recommend that such sites receive priority and stronger support from international decision makers and multilateral agreements for sampling. Although appealing in terms of for example, logistic and sampling comprehensiveness, sampling smaller sites may thus be of limited use with regard to population size and trend (but see Amano et al. 2018), especially when scheme resource allocation could allow to switch sampling effort from several sites with smaller counts to fewer sites with higher counts. This is not necessarily problematic, as a major sampling effort reallocation over the flyway could likely be modeled adequately. Indeed, with the exception of LORI, all other models were relatively insensitive to such a strongly “Unbalanced” design regarding site importance (despite optimized GLMM convergence issues).

## Author Contributions

**U. Godeau:** conceptualization (equal), formal analysis (equal), investigation (equal), methodology (equal), writing – original draft (equal), writing – review and editing (equal). **E. Gaget:** conceptualization (equal), formal analysis (equal), investigation (equal), methodology (equal), writing – original draft (equal), writing – review and editing (equal). **L. Dami:** conceptualization (equal), funding acquisition (equal), project administration (equal), validation (equal), writing – review and editing (equal). **K. Baddour:** data curation (equal), validation (equal), writing – review and editing (equal). **D. O. S. O. Daf:** data curation (equal), validation (equal), writing – review and editing (equal). **M. Dakki:** data curation (equal), validation (equal), writing – review and editing (equal). **T. Frost:** data curation (equal), validation (equal), writing – review and editing (equal). **M. Hornman:** data curation (equal), validation (equal), writing – review and editing (equal). **H. Kolberg:** data curation (equal), validation (equal), writing – review and editing (equal). **S.‐H. Lorentsen:** data curation (equal), validation (equal), writing – review and editing (equal). **B. Molina:** data curation (equal), validation (equal), writing – review and editing (equal). **F. E. F. F. Moniz:** data curation (equal), validation (equal), writing – review and editing (equal). **P. Defos du Rau:** conceptualization (equal), formal analysis (equal), funding acquisition (equal), investigation (equal), methodology (equal), project administration (equal), writing – original draft (equal), writing – review and editing (equal).

## Funding

This work was supported by the project “Innovations for migratory bird monitoring along the East Atlantic Flyway – FLYWAY” funded by DG REFORM, the Wadden Sea Flyway Initiative (Sovon/WSFI) and EUCC – The Coastal Union Germany (EUCC‐D).

## Disclosure

Statement on inclusion: Our study brings together authors from different countries, including scientists based in one country where the study was carried out.

## Conflicts of Interest

The authors declare no conflicts of interest.

## Supporting information


**Appendix S1:** ece372902‐sup‐0001‐AppendixS1.docx.

## Data Availability

All the required data are uploaded as Supporting Information S1.
