# Supplementary material for: Recommendations for Improving the Modeling of Wintering Waterbird Population Sizes and Trends
Source: Ecol Evol. 2026 Feb 17;16(2):e72902. doi: 10.1002/ece3.72902 (PMC12912885; doi:10.1002/ece3.72902)
Supplement: Supplementary file 1 — Appendix S1: ece372902‐sup‐0001‐AppendixS1.docx. [file ECE3-16-e72902-s001.docx]

Table SI.1: Countries included in the dataset and their geographical region used as predictor in TRIM models

| Countries | Geographical regions |
| --- | --- |
| Namibia, Senegal, South Africa | Sub-Saharan Africa |
| Morocco, Mauritania | North Africa |
| Germany, Denmark, Estonia, Belgium Flanders, Finland, United Kingdom of Great Britain and Northern Ireland, Ireland, Lithuania, Latvia, Netherlands, Norway, Sweden | North Europe |
| Spain, France, Portugal | South Europe |


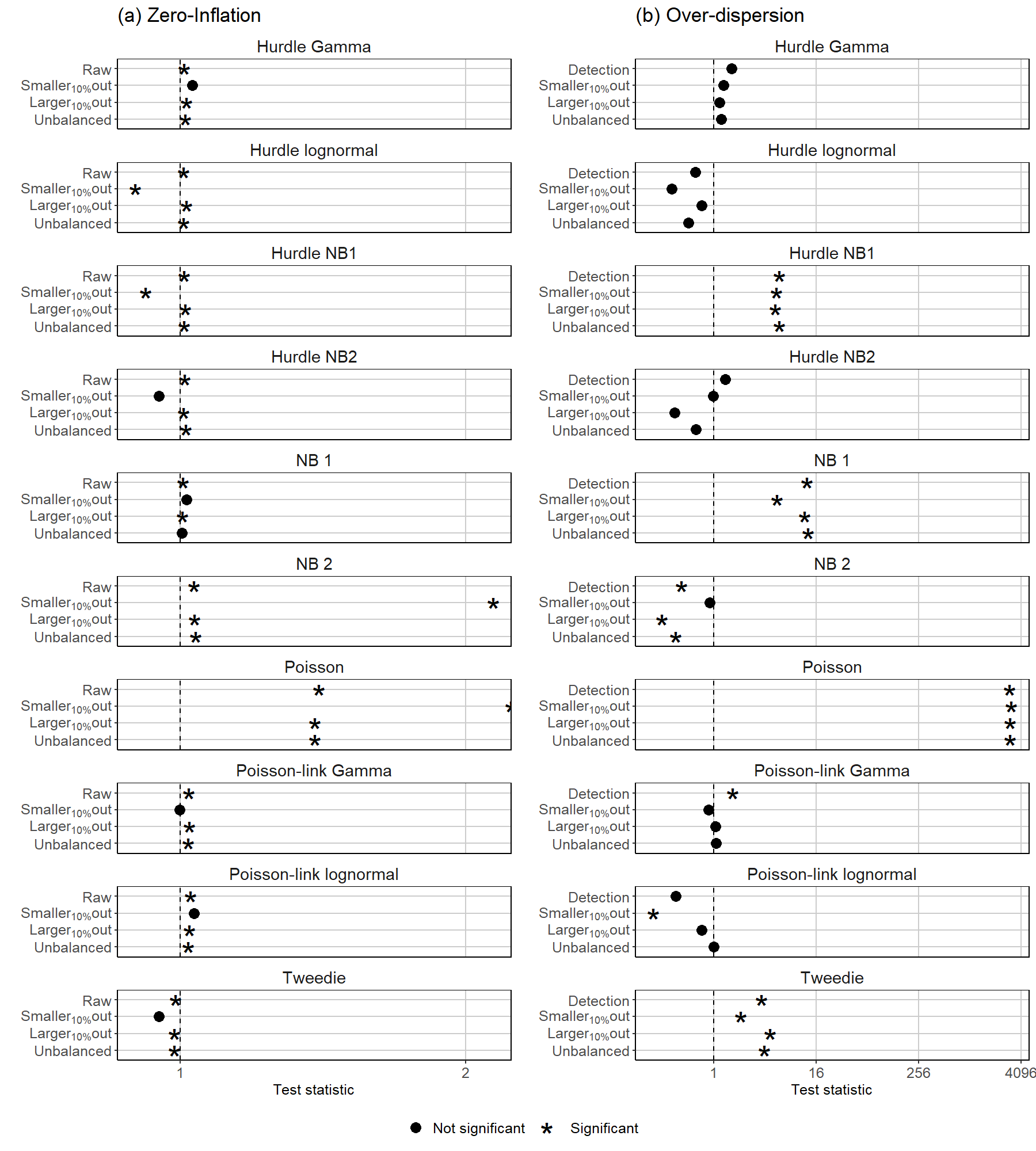


Figure SI 1: Zero-inflation (a) and dispersion (b) test values and significance for GLMM with the distribution families applied on the four different sampling designs for species A.


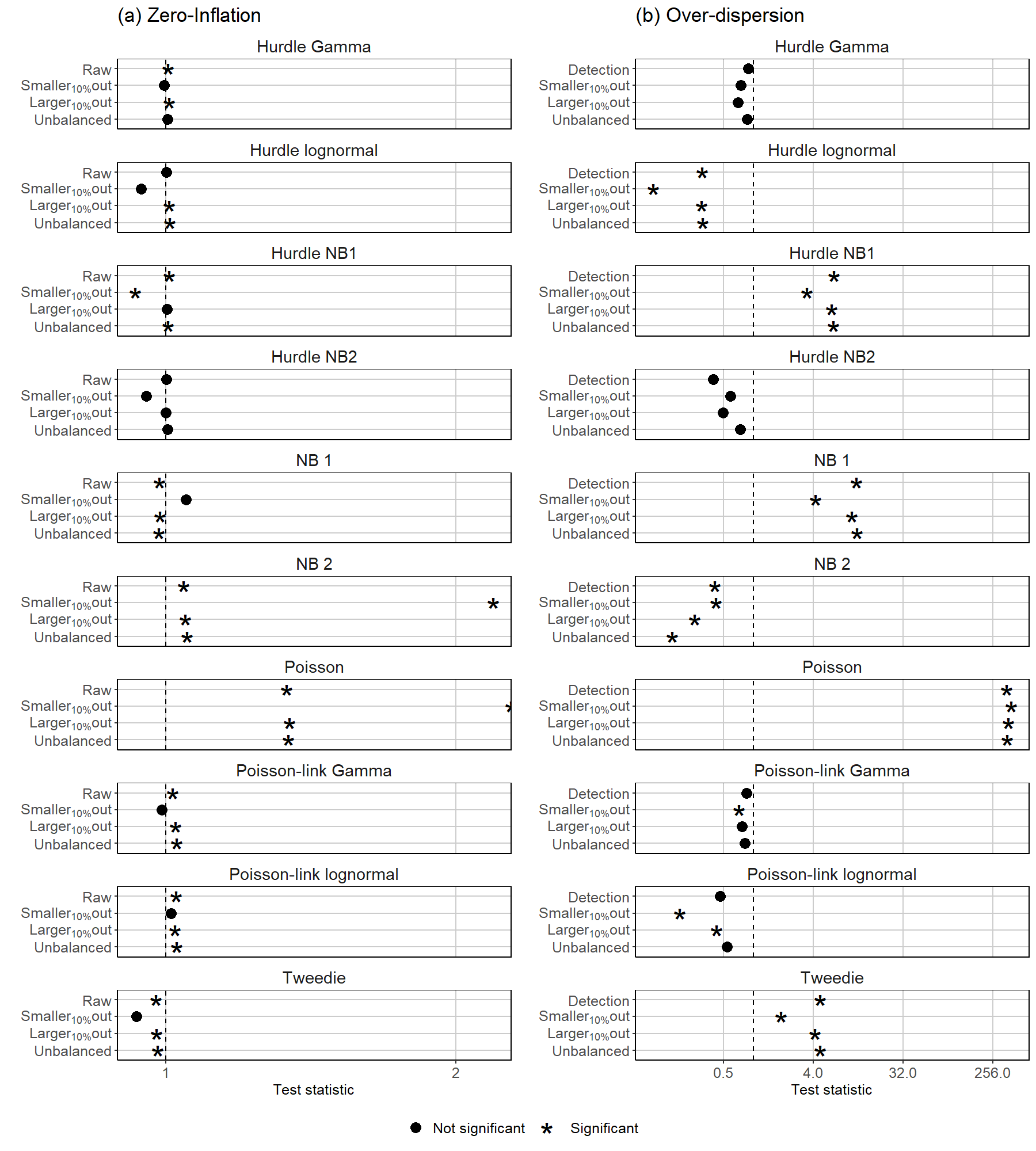


Figure SI 2: Zero-inflation (a) and dispersion (b) test values and significance for GLMM with the distribution families applied on the four different sampling designs for species B.


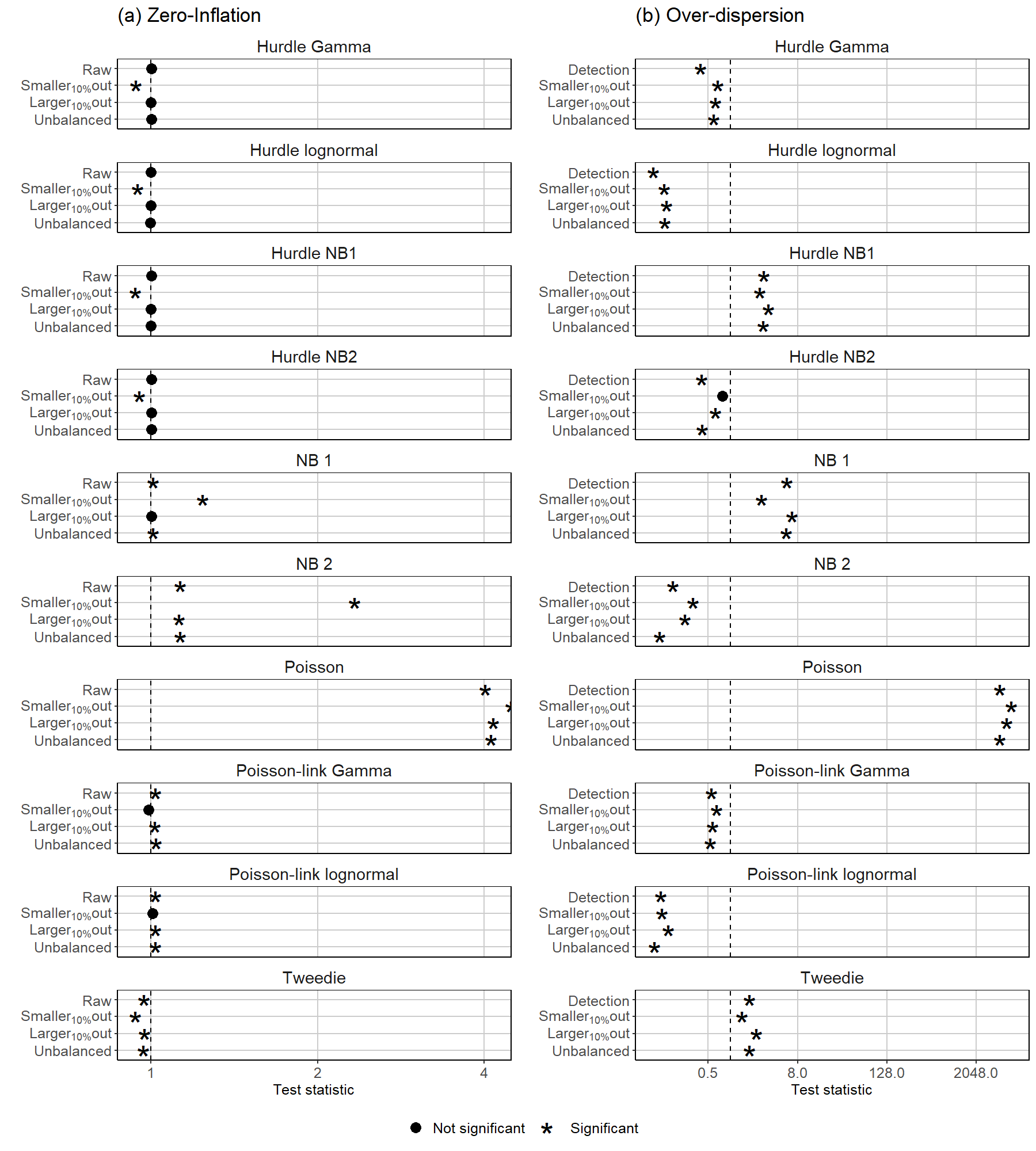


Figure SI 3: Zero-inflation (a) and dispersion (b) test values and significance for GLMM with the distribution families applied on the four different sampling designs for species C.


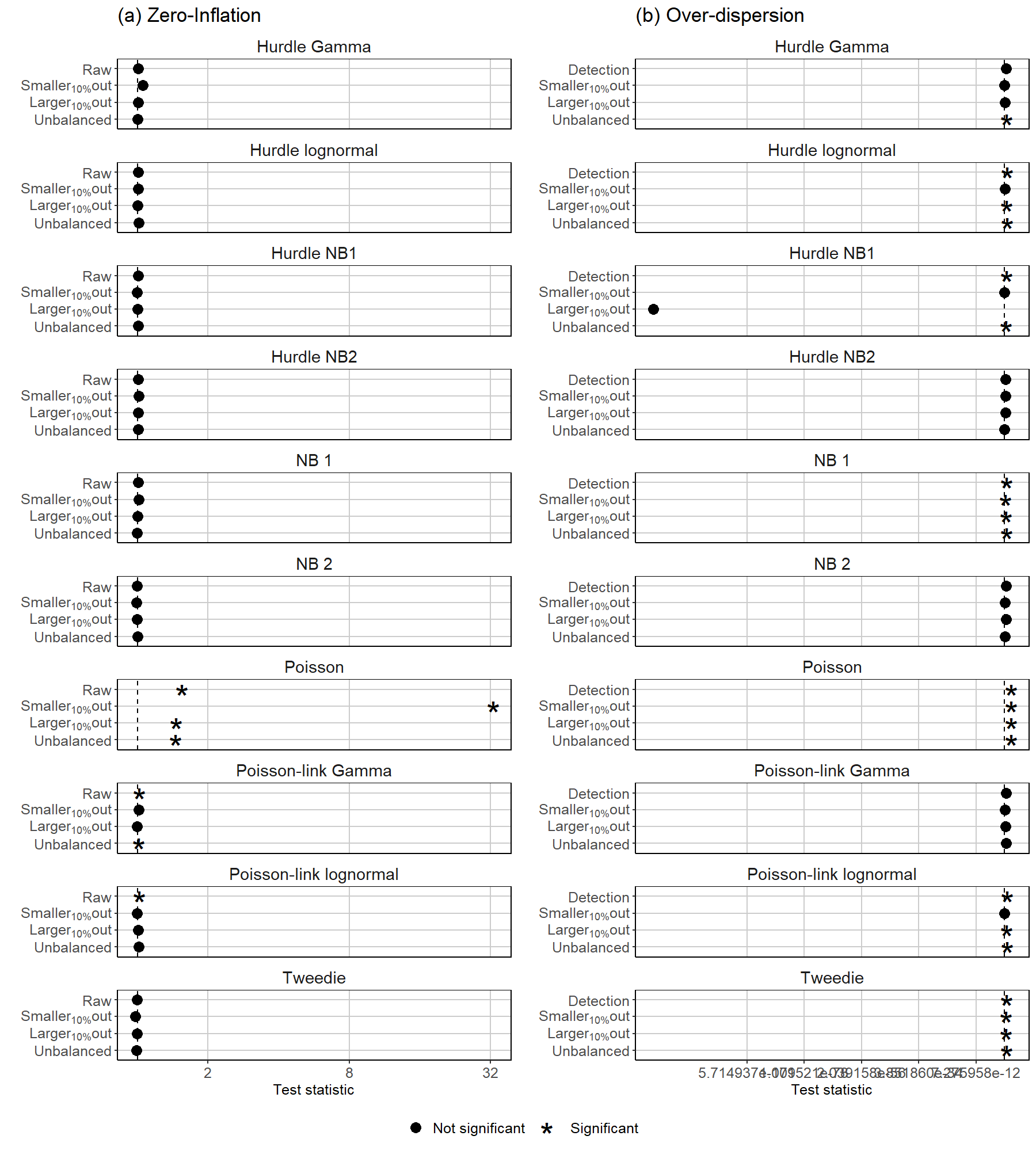


Figure SI 4: Zero-inflation (a) and dispersion (b) test values and significance for GLMM with the distribution families applied on the four different sampling designs for species D.


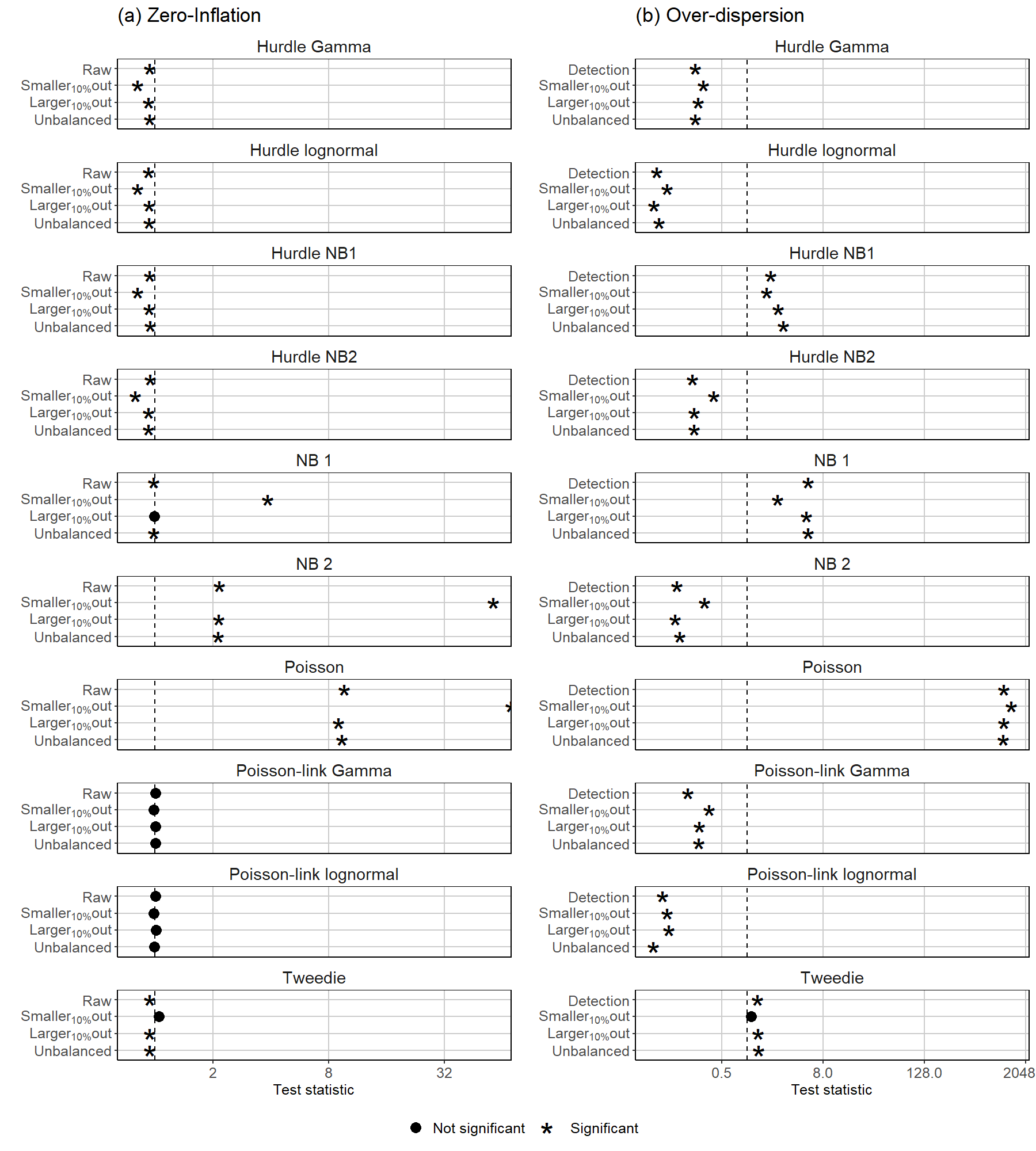


Figure SI 5: Zero-inflation (a) and dispersion (b) test values and significance for GLMM with the distribution families applied on the four different sampling designs for species D.


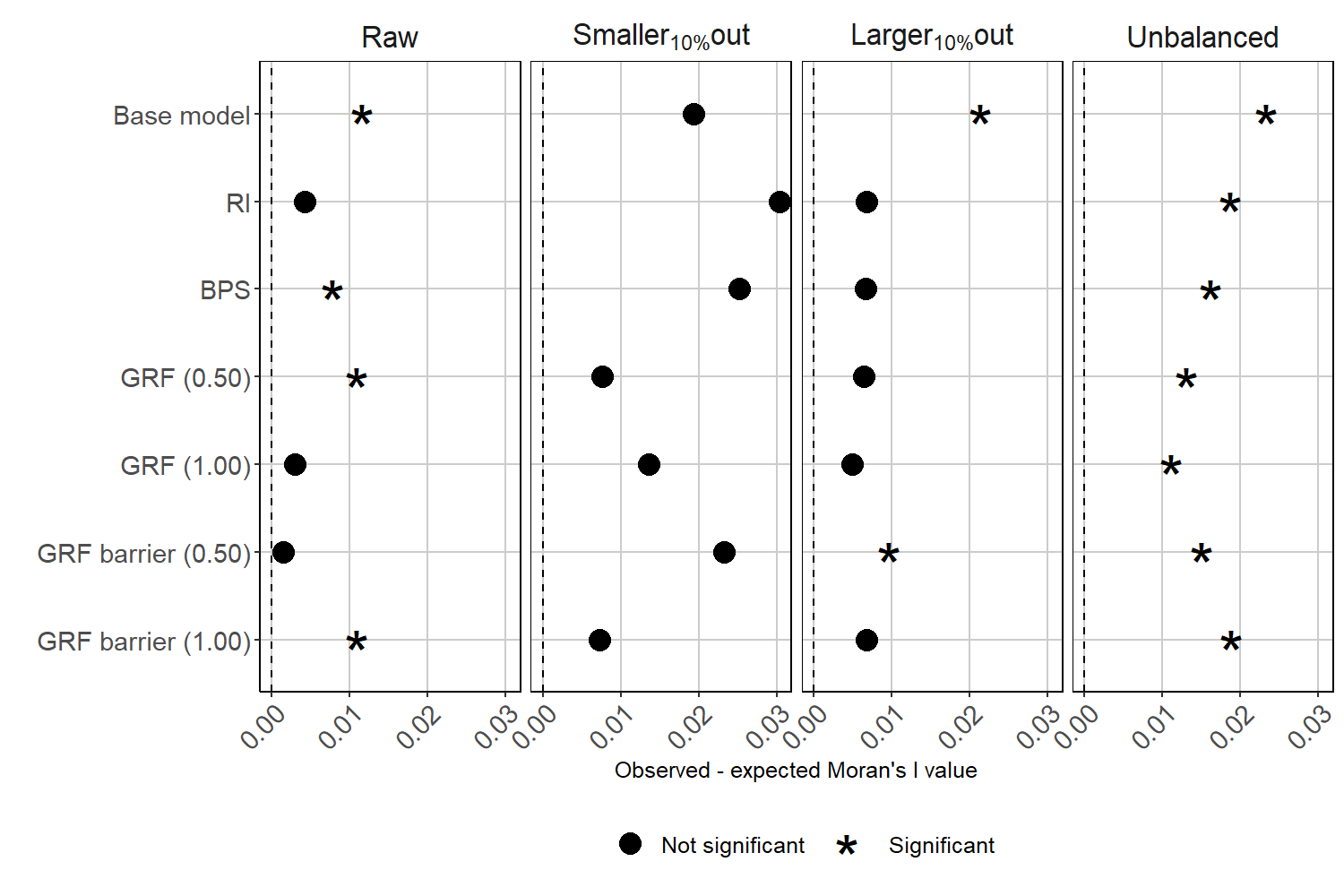


Figure SI 6: Difference between observed and expected Moran's I value and test significance of the GLMM with base specifications (Base model), Random intercept (RI), Gaussian random field (GRF), Basis penalty smoothing (BPS), with mesh in parenthesis when relevant, applied on the four different sampling designs for species A.


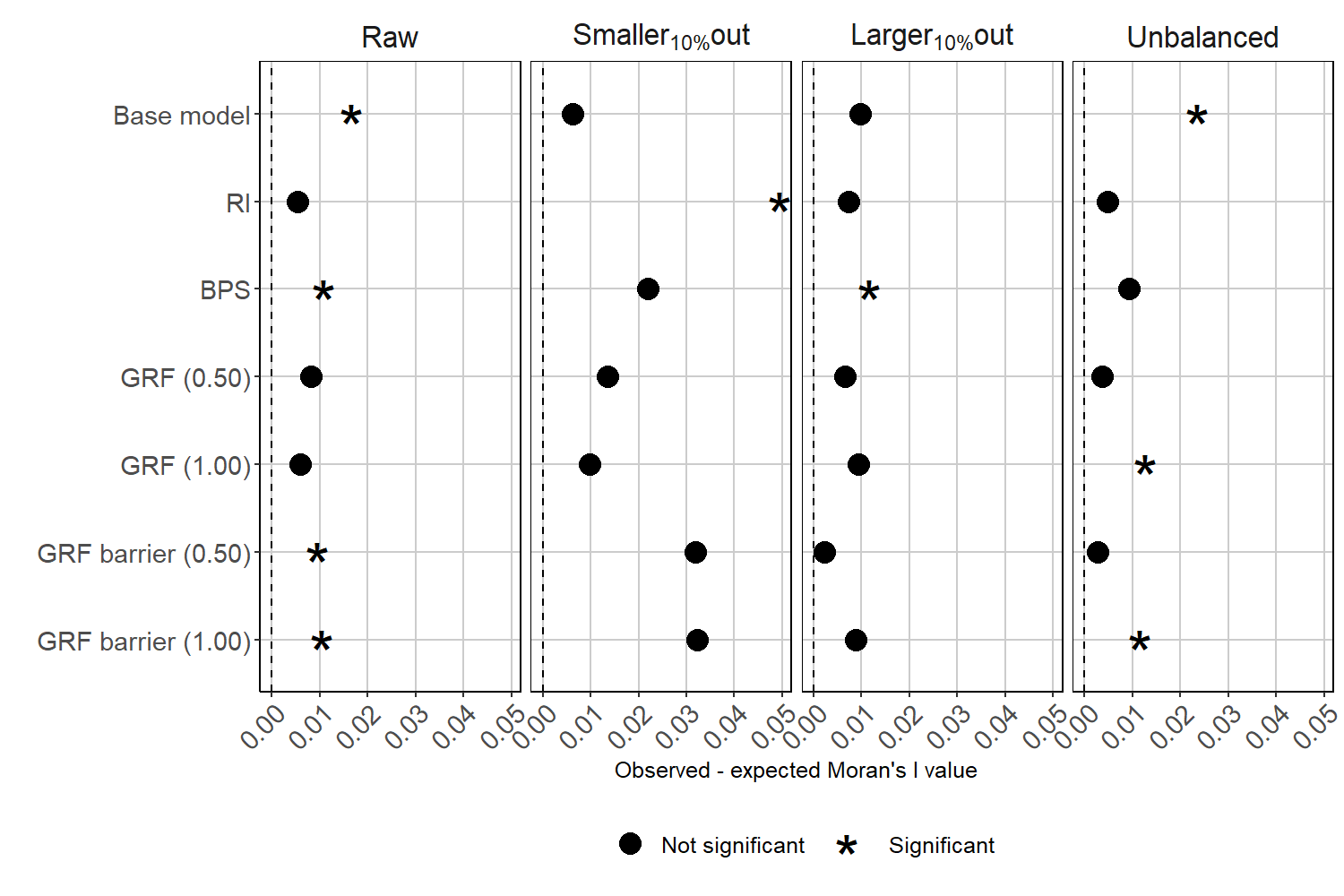


Figure SI 7: Difference between observed and expected Moran's I value and test significance of the GLMM with base specifications (Base model), Random intercept (RI), Gaussian random field (GRF), Basis penalty smoothing (BPS), with mesh in parenthesis when relevant, applied on the four different sampling designs for species B.


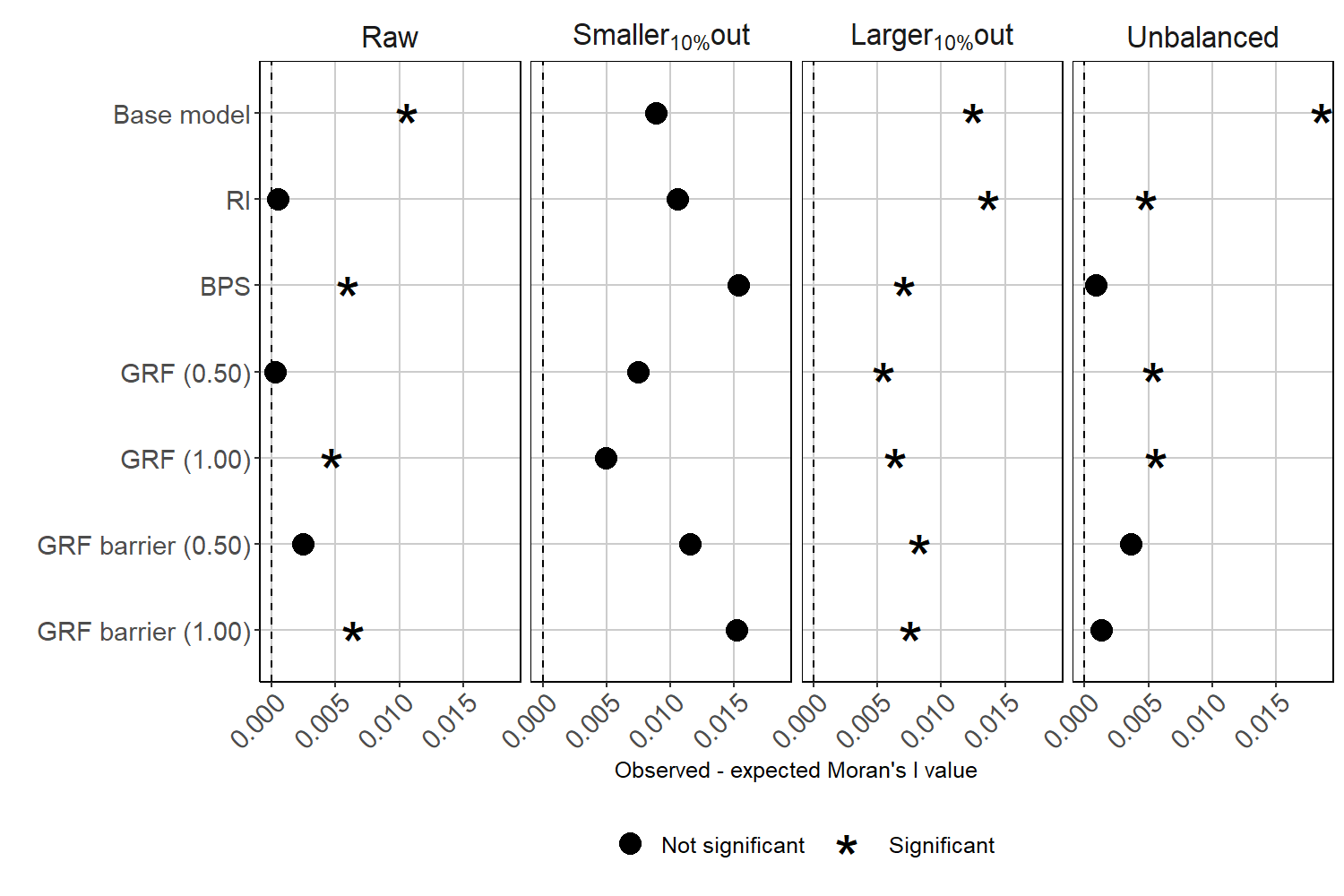


Figure SI 8: Difference between observed and expected Moran's I value and test significance of the GLMM with base specifications (Base model), Random intercept (RI), Gaussian random field (GRF), Basis penalty smoothing (BPS), with mesh in parenthesis when relevant, applied on the four different sampling designs for species C.


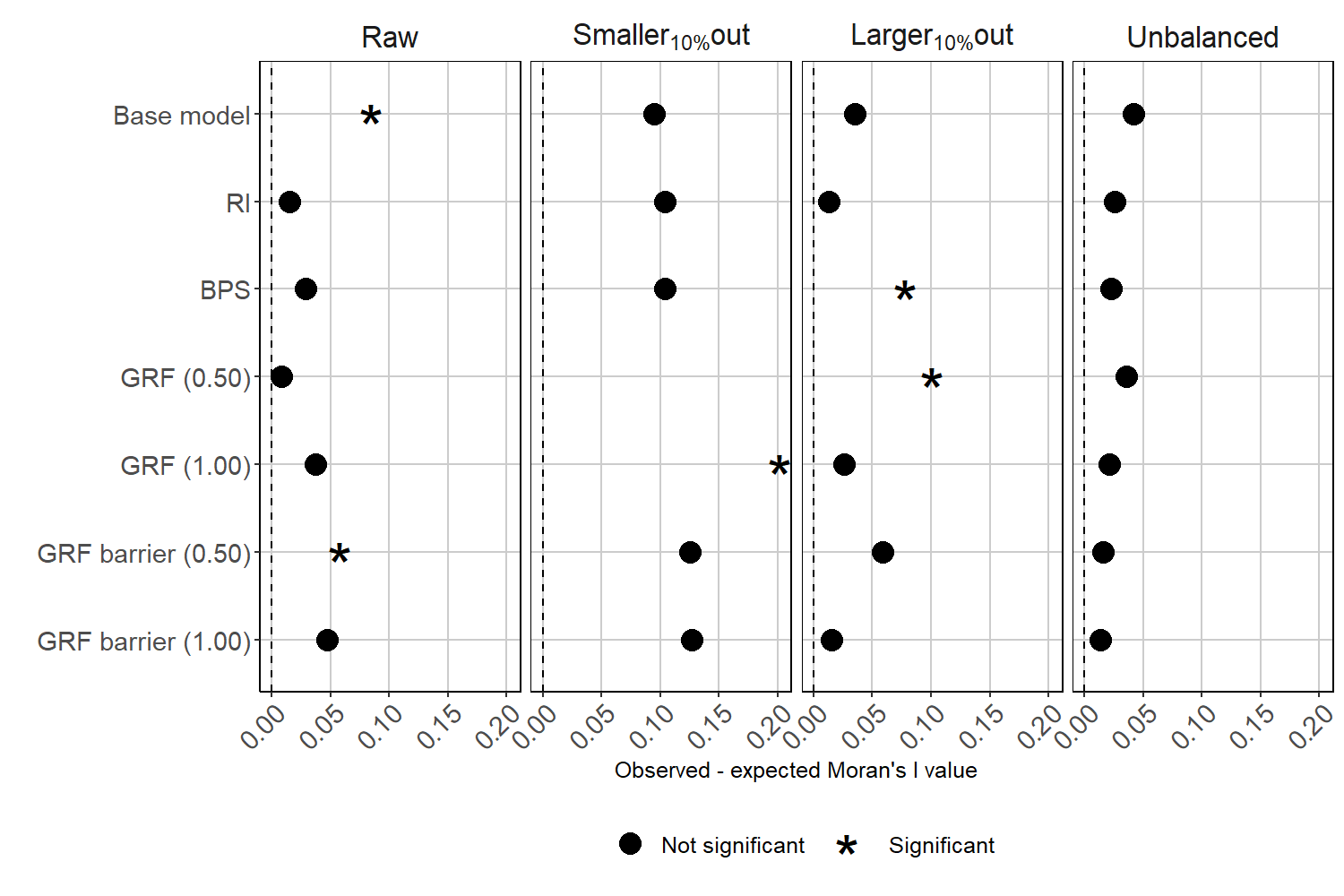


Figure SI 9: Difference between observed and expected Moran's I value and test significance of the GLMM with base specifications (Base model), Random intercept (RI), Gaussian random field (GRF), Basis penalty smoothing (BPS), with mesh in parenthesis when relevant, applied on the four different sampling designs for species D.


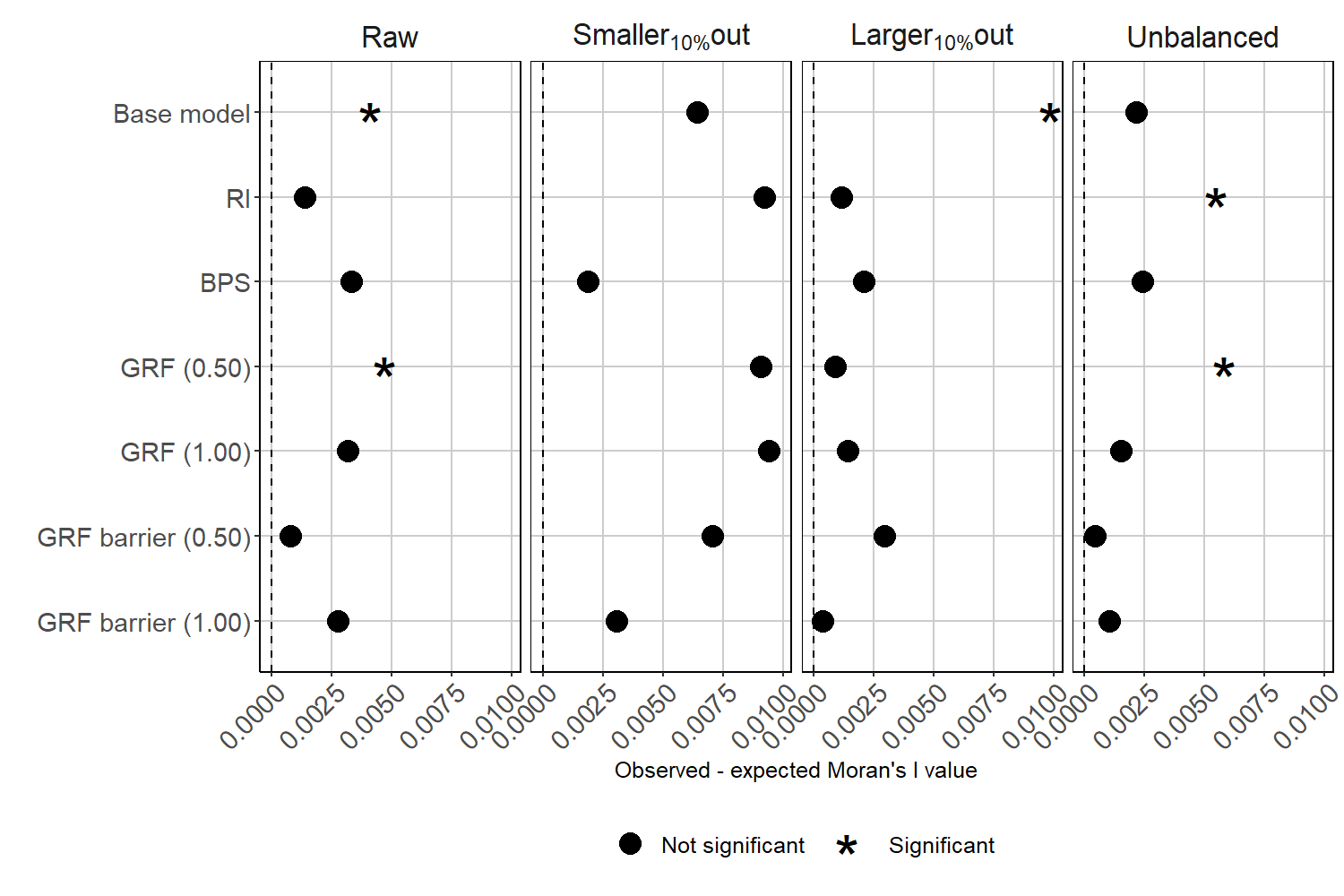


Figure SI 10: Difference between observed and expected Moran's I value and test significance of the GLMM with base specifications (Base model), Random intercept (RI), Gaussian random field (GRF), Basis penalty smoothing (BPS), with mesh in parenthesis when relevant, applied on the four different sampling designs for species E.


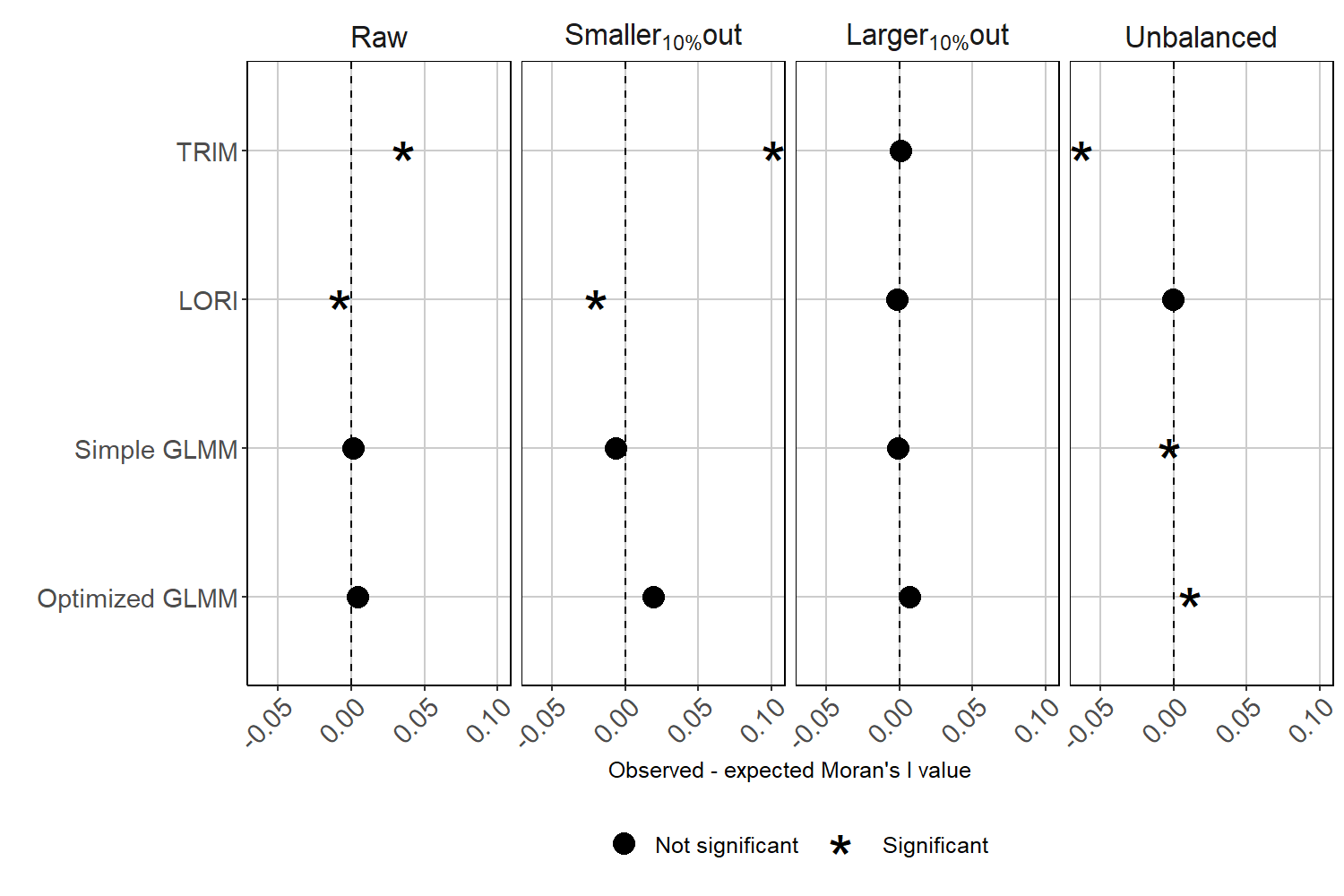


Figure SI 11 : Difference between observed and expected Moran's I value and test significance of the default setting GLMM, optimized GLMM, TRIM and LORI imputation models applied on the four different sampling designs for species A.


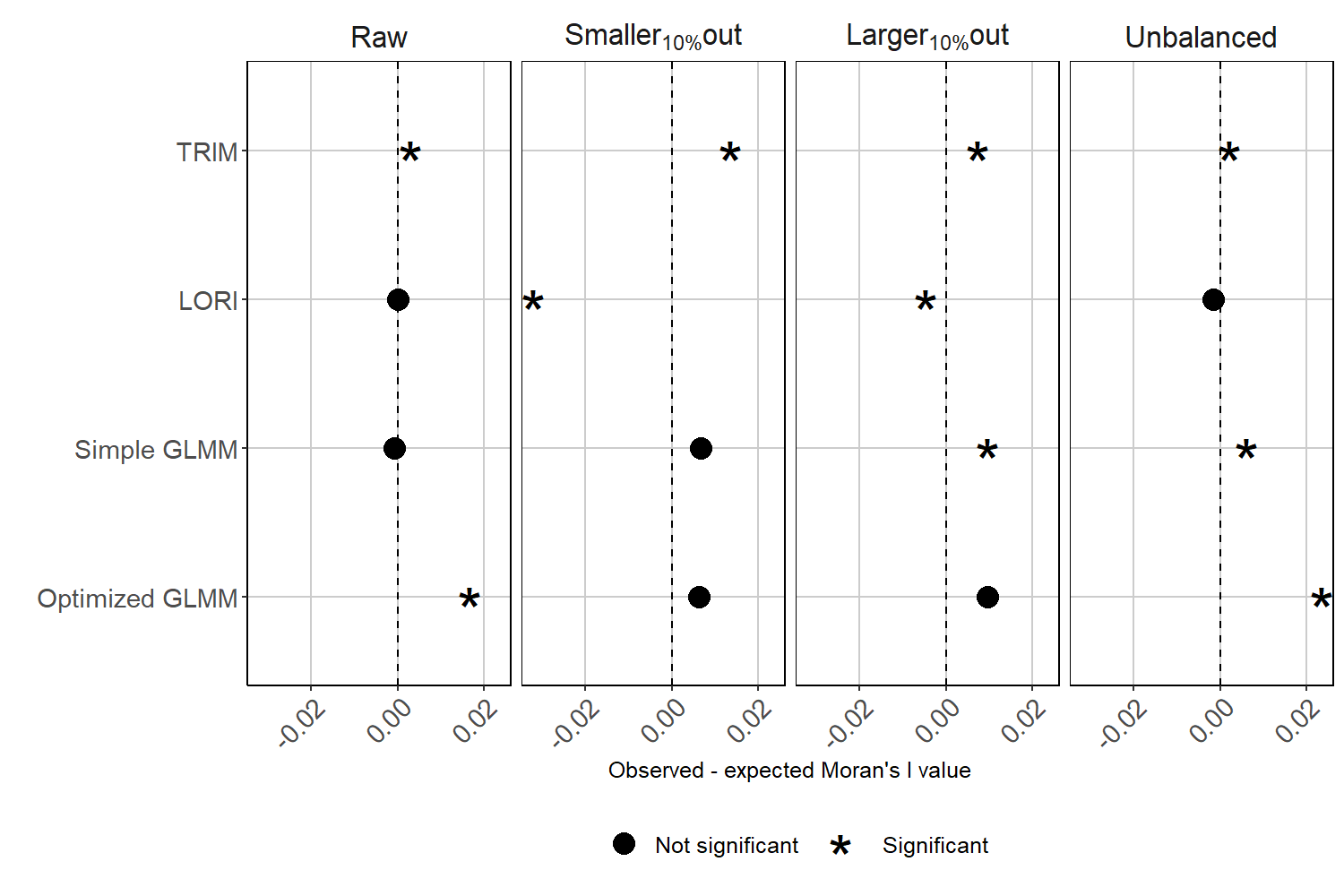


Figure SI 12: Difference between observed and expected Moran's I value and test significance of the default setting GLMM, optimized GLMM, TRIM and LORI imputation models applied on the four different sampling designs for species B.


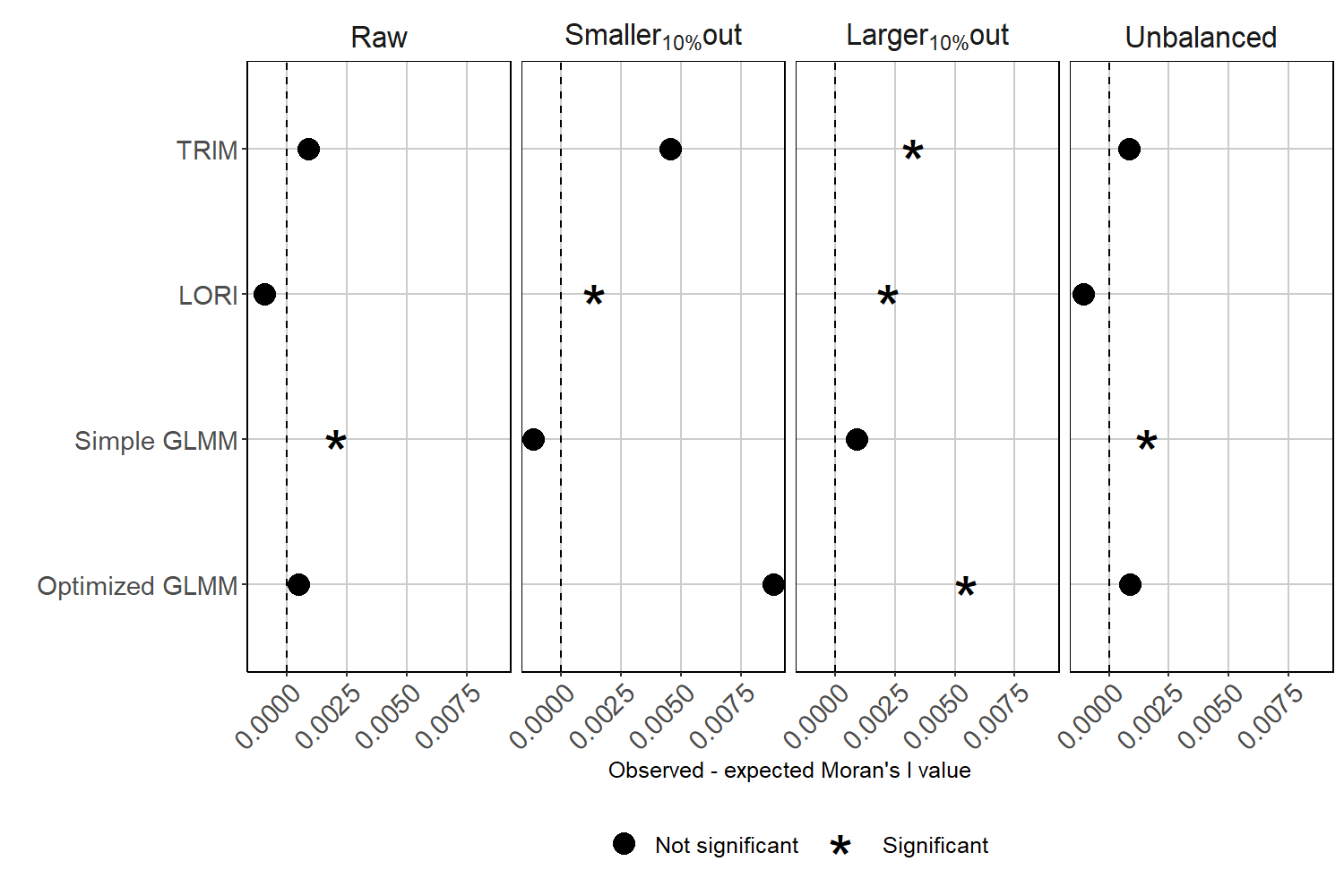


Figure SI 13: Difference between observed and expected Moran's I value and test significance of the default setting GLMM, optimized GLMM, TRIM and LORI imputation models applied on the four different sampling designs for species C.


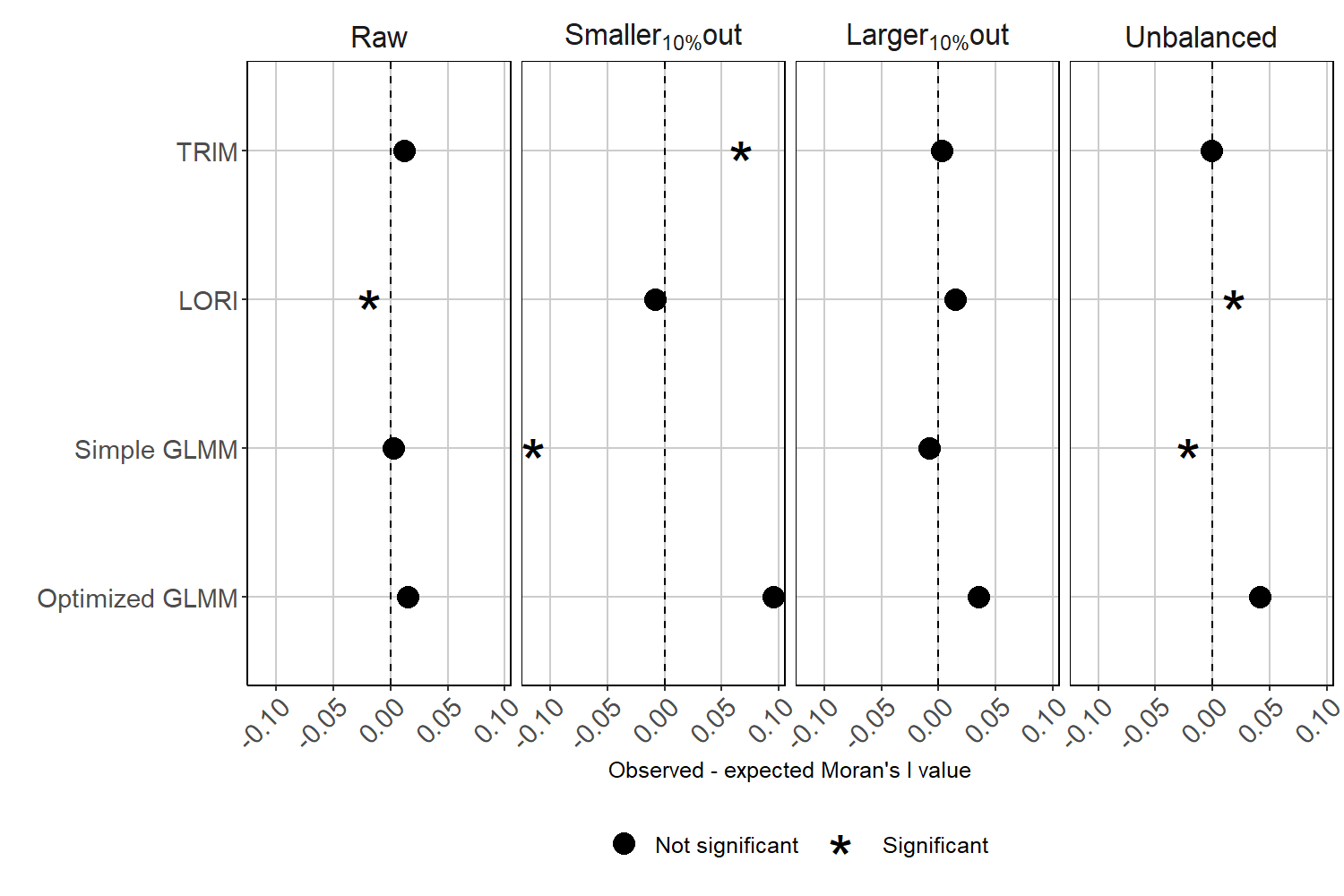


Figure SI 14: Difference between observed and expected Moran's I value and test significance of the default setting GLMM, optimized GLMM, TRIM and LORI imputation models applied on the four different sampling designs for species D.


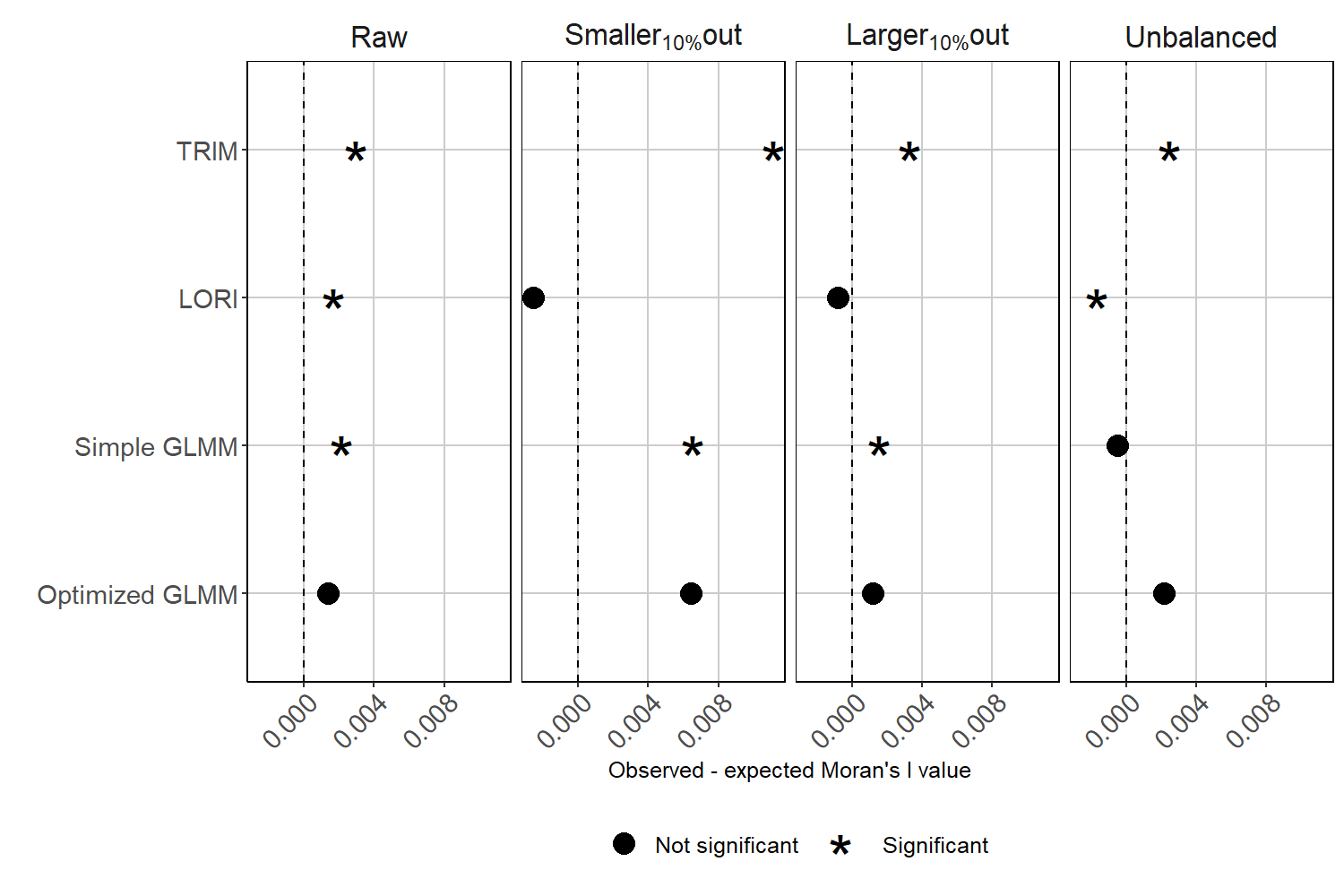


Figure SI 15: Difference between observed and expected Moran's I value and test significance of the default setting GLMM, optimized GLMM, TRIM and LORI imputation models applied on the four different sampling designs for species E.

Methods for detection of remaining zero-inflation

**DHARMa method**

The DHARMa method relies on multiple imputations, highlighting the proportion of instances in which discrepancies arise between observed and fitted data in terms of zero counts. Test statistics and p-values are estimated as:

test_statistic <- observed_zeros_nb / mean(fitted_zeros_nb)

test_pvalue <- min(min(mean(fitted_zeros_nb <= observed_zeros_nb),

mean(fitted_zeros_nb >= observed_zeros_nb)) * 2, 1)

With observed_zeros_nb and fitted_zeros_nb, the number of zeros in respectively observed or fitted data (excluding NA). In scenarios where 50% of cases (among multiple imputations) show more zeros in the observed data and 50% exhibit more zeros in the imputed data, the resulting p-value will be 1.0, indicating no significant difference. Conversely, if all cases reflect a higher count of zeros in either the observed or imputed data, the p-value will drop to 0.0. For intermediate situations, the p-value will vary continuously between 0 and 1, reflecting the degree of discrepancy between the datasets. Consequently, if we rely on a single imputation, as is the case with TRIM, this method becomes less relevant. It yields p-values of either 0.0 or 1.0, lacking nuance and merely addressing the question of whether there are more zeros or fewer, without providing any insight into the subtleties of the data.

**Chi-square method**

Conversely, the chi-square method is applicable in the case of a single imputation. The goal is to compare the frequency of zeros in the observed data with the frequency of zeros in the fitted matrix. It relies on a chi-square test that uses a contingency table of zero proportions, structured as follows:

|  | Zeros (Observed Data) | Non-Zeros (Observed Data) |
| --- | --- | --- |
| Zeros (Fitted Data) |  |  |
| Non-Zeros (Fitted Data) |  |  |

The test statistics and p-values are estimated as follows:

test_statistic <- (observed_zeros_freq - fitted_zeros_freq) / fitted_zeros_freq

test_pvalue <- chisq.test(table_contingency).

With observed_zeros_freq and fitted_zeros_freq the frequence of zeros in respectively observed or fitted data (excluding NA), and table_contigency the table of contingency as presented above.


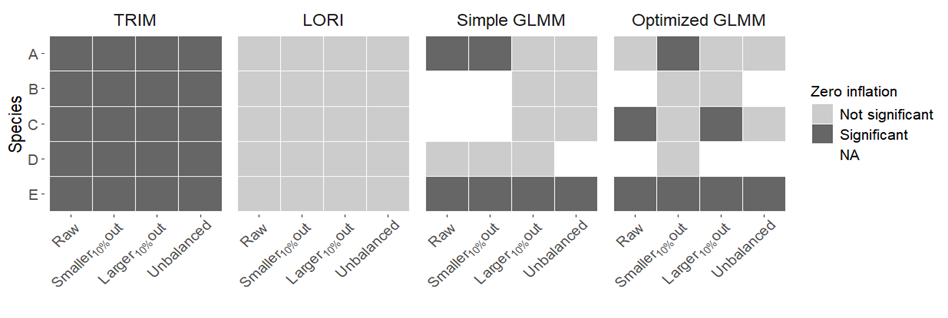


**Conclusion**

In general, the DHARMa test demonstrates greater sophistication in detecting zero-inflation and is more widely utilized for models akin to ours. Conversely, if the frequencies within the dataset do not exhibit significant differences, the chi-square test may fail to detect any inflation. The chi-square test is limited to identifying only substantial discrepancies in frequencies. Thus, the DHARMa test proves to be more nuanced in detecting zero-inflation, whereas the Chi-squared test may overlook inflation when the frequencies in the dataset are not sufficiently distinct. For example, in the context of the LORI models (as well as a simple GLMM), we observe that the DHARMa test consistently identifies zero-inflation, while the chi-square test does not indicate any such inflation. This suggests that, with the LORI model, the adjusted number of zeros is consistently insufficient compared to the observed zeros; however, the discrepancy in the number of zeros is not substantial. Consequently, the DHARMa method appears to be more sensitive to the detection of zero-inflation, as supported by its statistical framework and our data findings. Therefore, if the chi-square method indicates widespread autocorrelation with TRIM, it is highly likely that the DHARMa method would reveal similar patterns if we had multiple imputations available.
